# Discovery of plant extracts that greatly delay yeast chronological aging and have different effects on longevity-defining cellular processes

**DOI:** 10.18632/oncotarget.7665

**Published:** 2016-02-24

**Authors:** Vicky Lutchman, Younes Medkour, Eugenie Samson, Anthony Arlia-Ciommo, Pamela Dakik, Berly Cortes, Rachel Feldman, Sadaf Mohtashami, Mélissa McAuley, Marisa Chancharoen, Belise Rukundo, Éric Simard, Vladimir I. Titorenko

**Affiliations:** ^1^ Department of Biology, Concordia University, Montreal, Quebec H4B 1R6, Canada; ^2^ Idunn Technologies Inc., Rosemere, Quebec J7A 4A5, Canada

**Keywords:** yeast, cellular aging, longevity, plant extracts, aging-delaying chemical compounds

## Abstract

We discovered six plant extracts that increase yeast chronological lifespan to a significantly greater extent than any of the presently known longevity-extending chemical compounds. One of these extracts is the most potent longevity-extending pharmacological intervention yet described. We show that each of the six plant extracts is a geroprotector which delays the onset and decreases the rate of yeast chronological aging by eliciting a hormetic stress response. We also show that each of these extracts has different effects on cellular processes that define longevity in organisms across phyla. These effects include the following: 1) increased mitochondrial respiration and membrane potential; 2) augmented or reduced concentrations of reactive oxygen species; 3) decreased oxidative damage to cellular proteins, membrane lipids, and mitochondrial and nuclear genomes; 4) enhanced cell resistance to oxidative and thermal stresses; and 5) accelerated degradation of neutral lipids deposited in lipid droplets. Our findings provide new insights into mechanisms through which chemicals extracted from certain plants can slow biological aging.

## INTRODUCTION

The budding yeast *Saccharomyces cerevisiae* is a unicellular eukaryote amenable to comprehensive molecular analyses [1–3]. The development of various methods of such analyses for *S. cerevisiae* has enabled to uncover mechanisms underlying complex biological processes within individual yeast cells and their populations [1, 4, 5]. In addition, *S. cerevisiae* has relatively short and easy measurable replicative and chronological lifespans [6–13]. Due to these beneficial properties as a model organism for studying mechanisms of aging and longevity, *S. cerevisiae* has been used for the discovery of genes that slow cellular aging and increase healthy lifespan not only in *S. cerevisiae* and other yeasts but also in multicellular eukaryotes [6, 7, 9, 11, 14–16]. Furthermore, using *S. cerevisiae* as a model organism for elucidating mechanisms of cellular aging, several nutrient- and energy-sensing signaling pathways have been revealed; these pathways coordinate an evolutionarily conserved array of longevity-defining cellular processes not only in *S. cerevisiae* and other yeasts but also in eukaryotes across phyla [9, 11, 17–20]. Moreover, *S. cerevisiae* has been a model organism employed for uncovering several low molecular weight molecules that slow aging and extend healthy lifespan in various multicellular eukaryotes [10, 21–27]. All these studies employing *S. cerevisiae* as a model organism have provided evidence that the main features of biological aging have been conserved in the course of evolution [6, 9, 11, 18, 21, 28–31].

Our research is aimed at using *S. cerevisiae* as a model organism to discover chemical compounds that can slow aging and delay the onset of age-related diseases in evolutionarily distant eukaryotic organisms. Some of such geroprotective compounds have been previously revealed in natural products extracted from certain plants [25, 32, 33]. As a first step towards uncovering novel aging-delaying chemical compounds of plant origin, we conducted a screen for plant extracts (PEs) that can extend yeast chronological lifespan (CLS). Our screen revealed six PEs that increase yeast CLS considerably more efficiently than any of the longevity-extending chemical compounds yet described. We show that each of these PEs decelerates yeast chronological aging and has different effects on several longevity-defining cellular processes.

## RESULTS

### A screen for PEs that can extend longevity of chronologically aging yeast

We screened a library of PEs for extracts that can increase yeast CLS. This library includes 35 different PEs of known origin and properties (Tables 1 and 2, respectively). To perform the screen for lifespan-extending PEs, we used a robust assay for measuring yeast CLS. This assay was similar to the one described previously [34], but the wild-type strain BY4742 was cultured in the synthetic minimal YNB medium initially containing 2% glucose (instead of the nutrient-rich YEP medium supplemented with 0.5% glucose). Yeast cells cultured on 2% glucose are not limited in calorie supply; these cells age chronologically under so-called non-caloric restriction (non-CR) conditions that accelerate aging in different yeast genetic backgrounds, including BY4742 [6, 10, 11].

**Table 1:** A list of plant extracts that have been used in this study

Abbreviated name	Botanical name	Plant part used	Commercial source
PE1	*Echinacea purpurea*	Whole plant	Idunn Technologies
PE2	*Astragalus membranaceous*	Root	Idunn Technologies
PE3	*Rhodiola rosea L.*	Root	Idunn Technologies
PE4	*Cimicifuga racemosa*	Root and rhizome	Idunn Technologies
PE5	*Valeriana officinalis L.*	Root	Idunn Technologies
PE6	*Passiflora incarnate L.*	Whole plant	Idunn Technologies
PE7	*Polygonum cuspidatum*	Root and rhizome	Idunn Technologies
PE8	*Ginkgo biloba*	Leaf	Idunn Technologies
PE9	*Zingiber officinale Roscoe*	Rhizome	Idunn Technologies
PE10	*Theobroma cacao L.*	Cacao nibs	Idunn Technologies
PE11	*Camellia sinensis L. Kuntze*	Leaf	Idunn Technologies
PE12	*Apium graveolens L.*	Seed	Idunn Technologies
PE13	*Scutellaria baicalensis*	Root	Idunn Technologies
PE14	*Euterpe oleracea*	Fruit	Idunn Technologies
PE15	*Withania somnifera*	Root and leaf	Idunn Technologies
PE16	*Phyllanthus emblica*	Fruit	Idunn Technologies
PE17	*Camellia sinensis*	Leaf	Idunn Technologies
PE18	*Pueraria lobata*	Root	Idunn Technologies
PE19	*Silybum marianum*	Seed	Idunn Technologies
PE20	*Eleutherococcus senticosus*	Root and stem	Idunn Technologies
PE21	*Salix alba*	Bark	Idunn Technologies
PE22	*Glycine max L.*	Bean	Idunn Technologies
PE24	*Calendula officinalis*	Flower	Idunn Technologies
PE25	*Salvia miltiorrhiza*	Root	Idunn Technologies
PE27	*Panax quinquefolium*	Root	Idunn Technologies
PE28	*Harpagophytum procumbens*	Root	Idunn Technologies
PE29	*Olea europaea L.*	Leaf	Idunn Technologies
PE30	*Gentiana lutea*	Root	Idunn Technologies
PE31	*Piper nigrum*	Fruit	Idunn Technologies
PE32	*Aesculus hippocastanum*	Seed	Idunn Technologies
PE33	*Mallus pumila Mill.*	Fruit	Idunn Technologies
PE34	*Fragaria spp.*	Fruit	Idunn Technologies
PE35	*Ribes nigrum*	Leaf	Idunn Technologies
PE36	*Dioscorea opposita*	Root	Idunn Technologies
PE37	*Cinnamomum verum*	Bark	Idunn Technologies

**Table 2:** Properties of plant extracts that have been used in this study

Abbreviated name	Properties
PE1	Extraction solvent: ethanol (75%)/water (25%). Extract ratio: 4/1. Composition: natural extract, maltodextrin.
PE2	Extraction solvent: denatured ethanol (70%)/water (30%). Extract ratio: 10/1. Composition: natural extract (40–50%), gum arabic (50–60%).
PE3	Extraction solvent: ethanol (60–80%)/water (40–20%). Extract ratio: 15–20/1. Composition: natural extract (80–100%), maltodextrin (0–20%).
PE4	Extract ratio: 6–8/1. Composition: natural extract (28–38%), maltodextrin (60–70%), tricalcium phosphate (0–5%).
PE5	Extraction solvent: denatured ethanol/water. Extract ratio: ∼ 6/1. Composition: natural extract, maltodextrin, silica (0–1%).
PE6	Extraction solvent: water (100%). Extract ratio: 4/1. Composition: natural extract, maltodextrin.
PE7	Extraction solvent: ethanol (80%)/water (20%). Extract ratio: 40/1. Composition: natural extract (90–100%), maltodextrin (0–10%).
PE8	Extraction solvent: ethanol/water. Extract ratio: 50/1. Composition: natural extract.
PE9	Extraction solvent: ethanol/water. Composition: natural extract (96%), gingerols (4%).
PE10	Natural powder/final product ratio: 2–3/1. Composition: natural powder.
PE11	Extraction solvent: ethyl acetate (90%)/water (10%). Extract ratio: 6/1. Composition: natural extract (100%).
PE12	Extraction solvent: ethanol (90%)/water (10%). Extract ratio: 8/1. Composition: natural extract, maltodextrin, modified starch, silica.
PE13	Extraction solvent: ethanol/water. Extract ratio: 4/1. Composition: natural extract.
PE14	Extraction solvent: ethanol/water. Extract ratio: 4/1. Composition: natural extract.
PE15	Extraction solvent: water. Extract ratio: 9/1. Composition: withanolide glycoside conjugates (10%), oligosaccharides (32%), free withanolides (0.5%).
PE16	Extraction solvent: water. Composition: hydrolysable tannins (> 60%), including Emblicanin–A, Emblicanin–B, Punigluconin, Pedunculagin.
PE17	Composition: tea polyphenols (> 90%), including epigallocatechin gallate (> 40%).
PE18	Composition: flavonoids (> 40%), including puerarin.
PE19	Extraction solvent: ethanol/water. Composition: silymarin (> 80%).
PE20	Extraction solvent: water. Composition: eleutheroside B + E (> 0.8%).
PE21	Extraction solvent: ethanol/water. Composition: salicin (> 25%).
PE22	Composition: isoflavones (40%).
PE24	Composition: lutein (> 5%).
PE25	Composition: tanshinones, isotanshinones, cryptotanshinone, isocryptotanshinone, dihydrotanshinone, hydroxytanshinones.
PE27	Composition: ginsenosides (10%, by HPLC–UV), quintozene–free.
PE28	Extraction solvent: ethanol/water. Extract ratio: 40/1. Composition: harpagosides (20%, by HPLC–UV).
PE29	Extraction solvent: ethanol (70%)/water (30%). Extract ratio: 5–10/1. Composition: natural extract, maltodextrin, silica (0.2%).
PE30	Composition: isogentisin (0.04%).
PE31	Extraction solvent: ethanol. Extract ratio: 10/1. Composition: piperine (> 90%).
PE32	Composition: aescin (20%).
PE33	Extraction solvent: ethanol (70%)/water (30%). Extract ratio: 120–130/1. Composition: natural extract (60–70%), maltodextrin (30–40%).
PE34	Extract ratio: 5/1. Composition: natural extract, including polyphenols (> 2%).
PE35	Extraction solvent: water. Composition: polyphenols (15%, by HPLC–UV).
PE36	Composition: diosgenine (> 16%, by HPLC–UV).
PE37	Extraction solvent: water. Composition: polyphenols (25%, by HPLC–UV).

**Figure 1:**
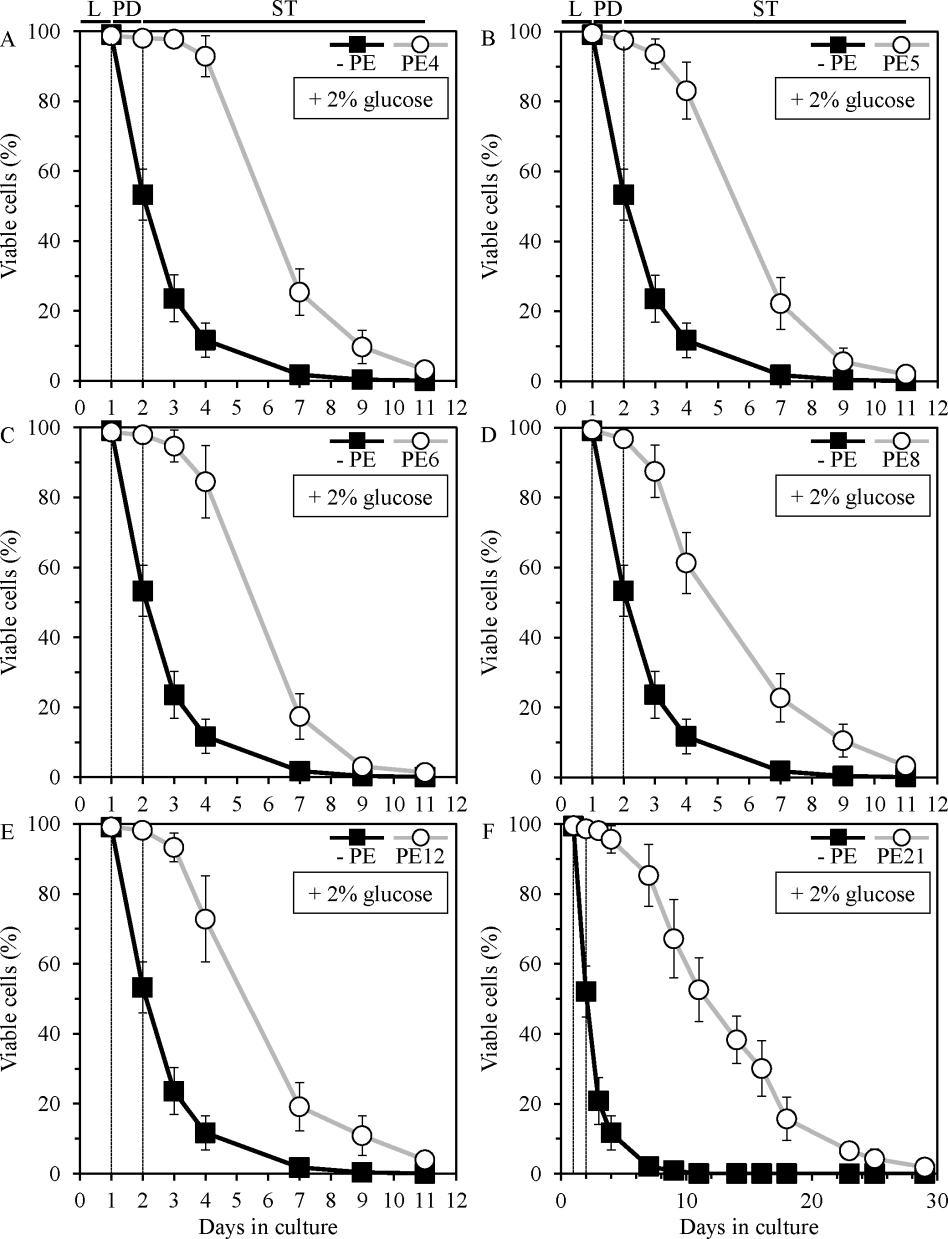
PE4, PE5, PE6, PE8, PE12 and PE21 extend the chronological lifespan (CLS) of yeast grown under non-caloric restriction (non-CR) conditions Wild-type (WT) cells were grown in the synthetic minimal YNB medium (0.67% Yeast Nitrogen Base without amino acids) initially containing 2% glucose, in the presence of a PE or in its absence. Survival curves of chronologically aging WT strain cultured with or without 0.5% PE4 (**A**), 0.5% PE5 (**B**), 1% PE6 (**C**), 0.3% PE8 (**D**), 0.1% PE12 (**E**) or 0.1% PE21 (**F**) are shown. Data are presented as means ± SEM (*n* = 21–35). CLS extension was significant for each of the PEs tested (*p* < 0.05; the *p* values for comparing survival curves were calculated with the help of the GraphPad Prism statistics software). Abbreviations: Logarithmic (L), post-diauxic (PD) or stationary (ST) growth phase.

In our screen for longevity-extending PEs, each PE from the library was added to growth medium at the time of cell inoculation at a final concentration in the 0.02% to 1.0% range. Some PEs from the library did not alter the mean and maximum CLS of yeast under non-CR conditions at any concentration examined; among these PEs were PE9, PE13, PE16, PE22, PE28 and PE36 (Figure S2–Figure S5). Many PEs from the library shortened the mean and/or maximum CLS of yeast under non-CR conditions at final concentrations ranging from 0.08% to 1.0%; among these PEs were PE1–PE3, PE7, PE10, PE11, PE14, PE15, PE17–PE20, PE24, PE25, PE27, PE29–PE35 and PE37 (Figure S1–Figure S5). 6 of the 35 PEs from the library significantly increased both the mean and maximum CLS of yeast under non-CR conditions if added at final concentrations ranging from 0.04% to 1.0% (Figure S1–Figure S3). A group of these longevity-extending PEs included the following extracts: 1) 0.5% PE4 from *Cimicifuga racemosa* (Figure 1A, Figure 3A and 3B, Figure S1); 2) 0.5% PE5 from *Valeriana officinalis L.* (Figure 1B, Figure 3A and 3B, Figure S1); 3) 1.0% PE6 from *Passiflora incarnata L.* (Figure 1C, Figure 3A and 3B, Figure S1); 4) 0.3% PE8 from *Ginkgo biloba* (Figure 1D, Figure 3A and 3B, Figure S1); 5) 0.1% PE12 from *Apium graveolens L.* (Figure 1E, Figure 3A and 3B, Figure S2); and 6) 0.1% PE21 from *Salix alba* (Figure 1F, Figure 3A and 3B, Figure S3). None of the six lifespan-prolonging PEs affected growth rates in logarithmic (L) and post-diauxic (PD) phases or impacted the maximum cell density in stationary (ST) phase of yeast cultures under non-CR conditions on 2% glucose (Figure S6). Thus, the observed lifespan extension by each of these PEs is unlikely to be caused by its ability to decrease growth rate or to make yeast more resistant to toxic substances accumulated during culturing in the synthetic minimal YNB medium.

**Figure 2:**
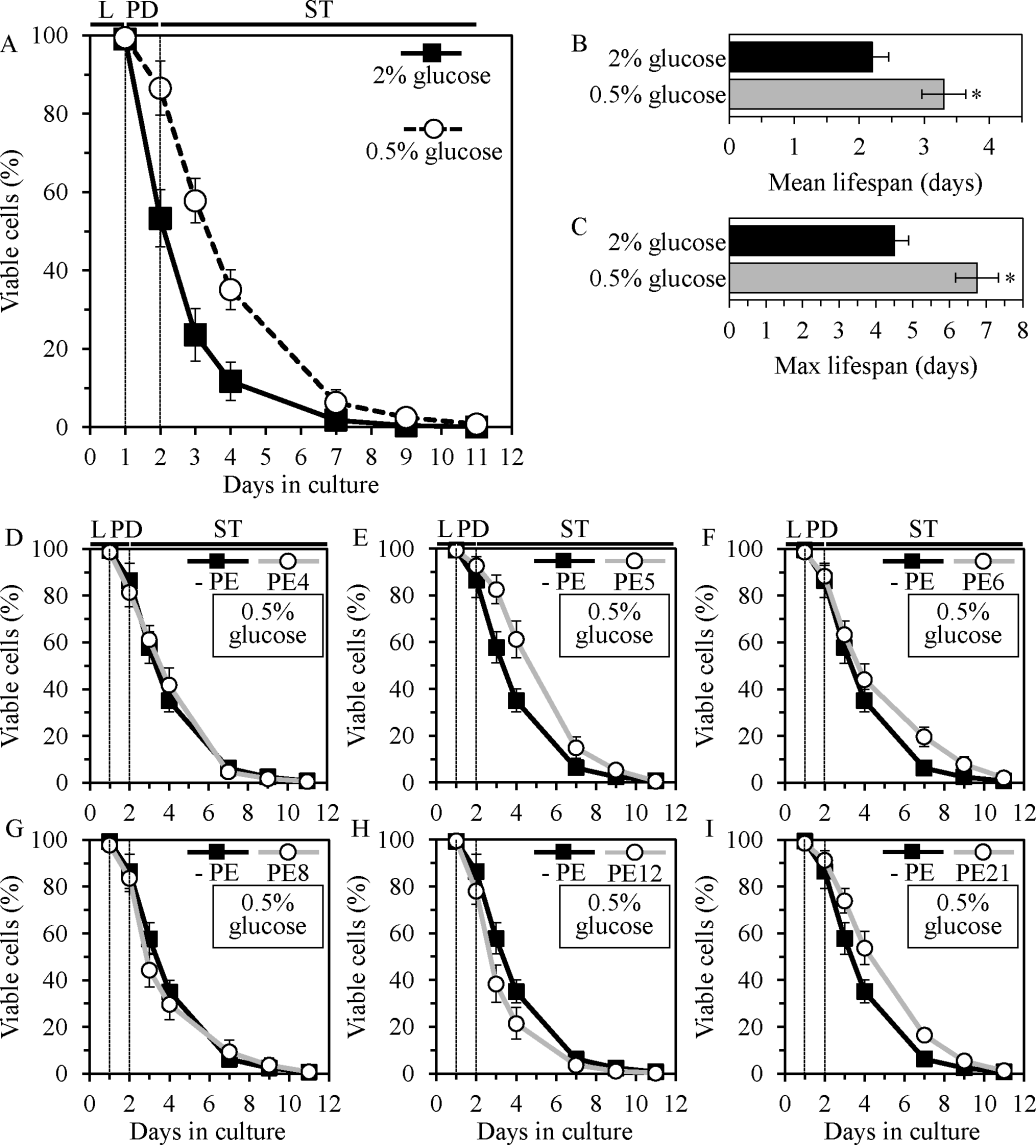
PE5 and PE21, but not PE4, PE6, PE8 or PE12, extend the CLS of yeast grown under CR conditions WT cells were grown in the synthetic minimal YNB medium initially containing 0.5% glucose (CR conditions) or 2% glucose (non-CR conditions), in the presence of a PE or in its absence. Survival curves (**A**), the mean (**B**) and maximum (**C**) lifespans of chronologically aging WT strain cultured under CR or non-CR conditions in the absence of a PE are shown; data are presented as means ± SEM (*n* = 5–7). CR caused significant extension of CLS (**A**) (*p* < 0.05; the *p* values for comparing survival curves were calculated with the help of the GraphPad Prism statistics software). CR extended both the mean (**B**) and maximum (**C**) lifespans (*p < 0.05; the *p* values for comparing the means of two groups were calculated with the help of the GraphPad Prism statistics software using an unpaired two-tailed *t* test). Survival curves of chronologically aging WT strain cultured under CR on 0.5% glucose with or without 0.5% PE4 (**D**), 0.5% PE5 (**E**), 1% PE6 (**F**), 0.3% PE8 (**G**), 0.1% PE12 (**H**) or 0.1% PE21 (**I**) are shown; data are presented as means ± SEM (*n* = 5–7). CLS extension under CR on 0.5% glucose was significant for PE5 and PE21 (*p* < 0.05; the *p* values for comparing survival curves were calculated with the help of the GraphPad Prism statistics software). CLS extension under CR on 0.5% glucose was not significant for PE4, PE6, PE8 and PE12. Abbreviations: Logarithmic (L), post-diauxic (PD) or stationary (ST) growth phase.

**Figure 3:**
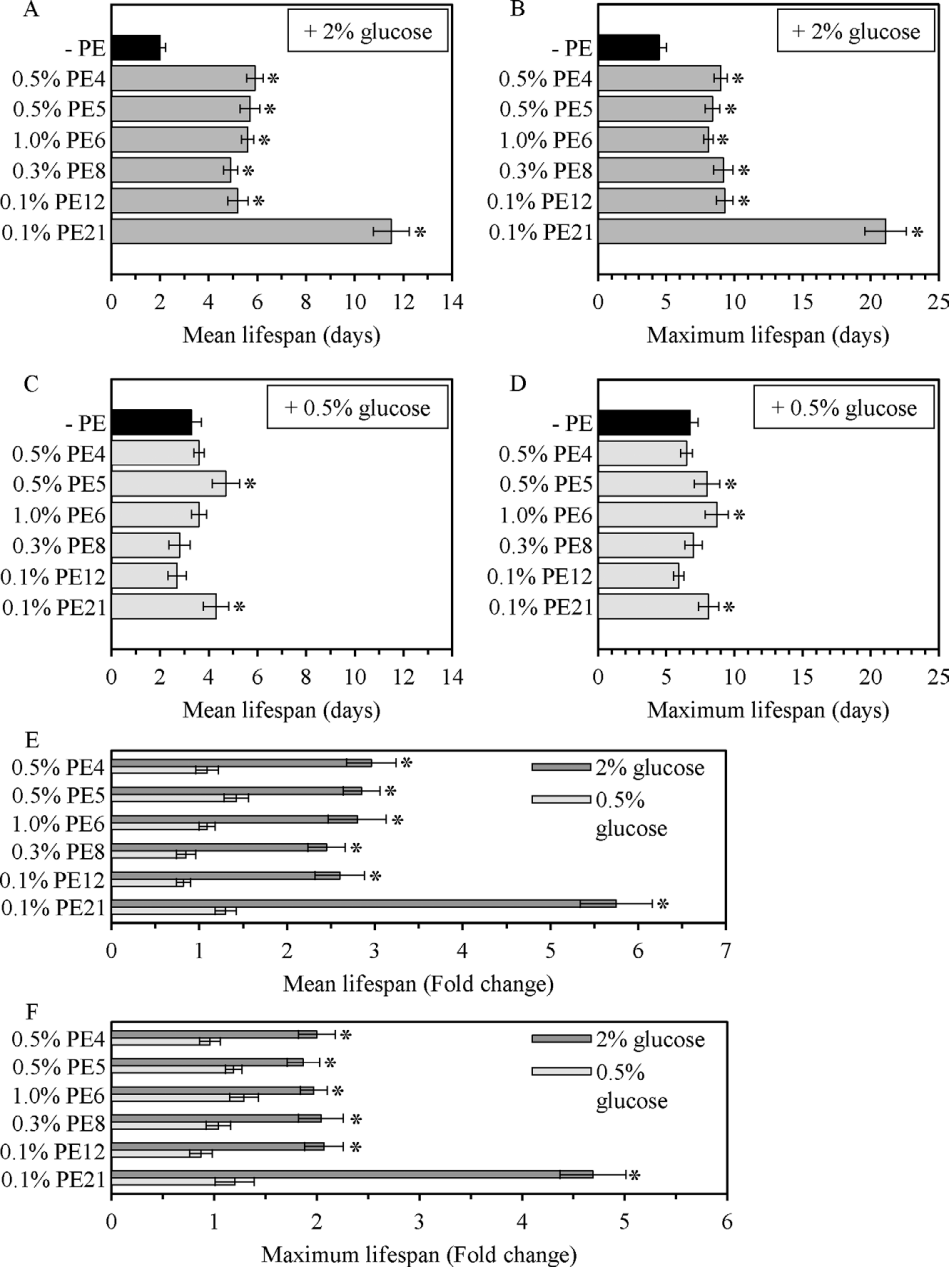
The longevity-extending efficacy under non-CR conditions significantly exceeds that under CR conditions for each of the six lifespan-prolonging PEs WT cells were grown in the synthetic minimal YNB medium initially containing 0.5% glucose (CR conditions) or 2% glucose (non-CR conditions), in the presence of a PE or in its absence. The mean (**A**, **C** and **E**) and maximum (**B**, **D** and **F**) lifespans of chronologically aging WT strain cultured under CR (**C**, **D**, **E** and **F**) or non-CR (**A**, **B**, **E** and **F**) conditions in the absence of a PE or in the presence of 0.5% PE4, 0.5% PE5, 1% PE6, 0.3% PE8, 0.1% PE12 or 0.1% PE21 are shown; data are presented as means ± SEM (*n =* 5–7; *p < 0.05). The extent to which each of the PE tested increases the mean and maximum lifespans under non-CR conditions exceeds that under CR conditions (*p < 0.05; the *p* values for comparing the means of two groups were calculated with the help of the GraphPad Prism statistics software using an unpaired two-tailed *t* test).

### For each of the six lifespan-prolonging PEs, the longevity-extending efficacy under CR conditions is significantly lower than that under non-CR conditions

Chronologically aging yeast grown under CR conditions on 0.5% glucose are known to live longer than yeast cultured under non-CR conditions on 2% glucose. Such ability of the CR diet to extend CLS has been reported for yeast cultured in media of various nutrient compositions [6, 10, 11]. We found that, if the CR diet is administered by culturing yeast in the YNB medium initially containing 0.5% glucose, it significantly increases both the mean and maximum CLS of *S. cerevisiae* (Figure 2A–2C). We discovered that 0.5% PE5 and 0.1% PE21 (but not 0.5% PE4, 1.0% PE6, 0.3% PE8 or 0.1% PE12) extend the mean CLS of yeast grown under CR conditions (Figure 2D–2I, Figure 3C). We also revealed that 0.5% PE5, 1.0% PE6 and 0.1% PE21 (but not 0.5% PE4, 0.3% PE8 or 0.1% PE12) extend the maximum CLS of yeast grown under CR conditions (Figure 2D–2I, Figure 3D). Akin to their effects under non-CR conditions, none of the six lifespan-prolonging PEs influenced growth rates in L and PD phases or altered the maximum cell density in ST phase of yeast cultures under CR conditions on 0.5% glucose (Figure S7). Importantly, each of the six lifespan-prolonging PEs extended both the mean and maximum CLS of yeast cultures under non-CR conditions on 2% glucose to a significantly higher extent than those of yeast under CR at 0.5% glucose (Figure 3A–3F). This observation indicates that each of these PEs could mimic the longevity-extending effect of CR.

### Each of the six longevity-extending PEs is a geroprotector which delays the onset and slows the progression of yeast chronological aging by eliciting a hormetic stress response

As we found, PE4, PE5, PE6, PE8, PE12 and PE21 greatly extend the mean CLS of yeast cultured under non-CR conditions (Figure 1A, Figure 3A and 3B, Figure S1–S3). Mean lifespan is believed to be directly proportional to the survival rates of organisms in the population during development and maturity stages of organismal aging; mean lifespan is likely to be under control of certain extrinsic (environmental) factors [35–38]. Thus, it is conceivable that PE4, PE5, PE6, PE8, PE12 and PE21 decrease the extrinsic rate of yeast chronological aging prior to cell entry into quiescence or senescence.

Furthermore, we revealed that PE4, PE5, PE6, PE8, PE12 and PE21 also substantially increase the maximum CLS of yeast grown under non-CR conditions (Figure 1A, Figure 3A and 3B, Figure S1–S3). Maximum lifespan is believed to reflect the duration of “healthy” life period (i.e. healthspan) during quiescence/senescence stage of organismal aging; maximum lifespan is likely to be controlled by certain intrinsic (cellular and organismal) longevity modifiers [7, 35–37, 39, 40]. One could therefore conclude that PE4, PE5, PE6, PE8, PE12 and PE21 also decrease the intrinsic rate of yeast chronological aging after cell entry into quiescence or senescence.

**Figure 4:**
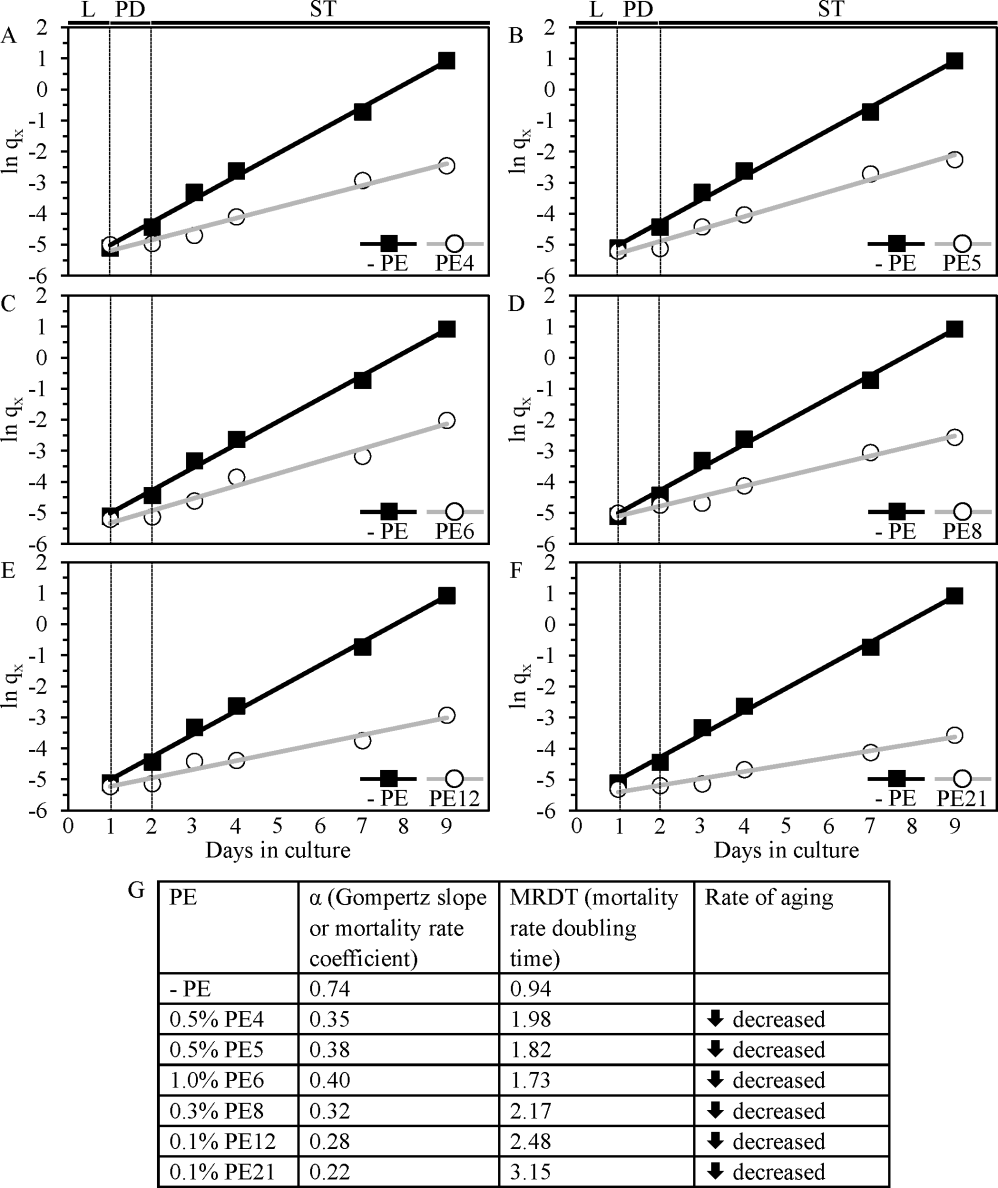
Analysis of the Gompertz mortality function indicates that PE4, PE5, PE6, PE8, PE12 and PE21 significantly decrease the rate of chronological aging in yeast WT cells were grown in the synthetic minimal YNB medium initially containing 2% glucose, in the presence of a PE or in its absence. Survival curves shown in Figure 1A–1F were used to calculate the age-specific mortality rates (q_x_) of chronologically aging WT yeast populations cultured with or without 0.5% PE4 (**A**), 0.5% PE5 (**B**), 1% PE6 (**C**), 0.3% PE8 (**D**), 0.1% PE12 (**E**) or 0.1% PE21 (**F**). Each of these longevity-extending PEs caused a substantial decrease in slope of the Gompertz mortality rate (also known as mortality rate coefficient α) and a considerable increase in the mortality rate doubling time (MRDT) (**G**). The values of q_x_, α and MRDT were calculated as described in Materials and methods. Abbreviations: Logarithmic (L), post-diauxic (PD) or stationary (ST) growth phase.

Our analysis of the Gompertz mortality function further validated the above conclusion that PE4, PE5, PE6, PE8, PE12 and PE21 significantly reduce the rate of yeast chronological aging. Indeed, we found that each of these longevity-extending PEs causes a substantial decrease in slope of the Gompertz mortality rate (also known as mortality rate coefficient α) and a considerable increase in the mortality rate doubling time (MRDT) (Figure 4A–4G). Such changes in the values of α and MRDT are characteristic of interventions that decrease the rate of progression through the process of biological aging [37, 41–44].

Noteworthy, our analyses of how different concentrations of PE4, PE5, PE6, PE8, PE12 and PE21 impact yeast longevity under non-CR conditions revealed that each of them causes a so-called “hormetic” stress response in chronologically aging yeast with respect to longevity. Indeed, the dose-response curves (i.e. the curves that reflect relationships between PE concentrations and mean or maximum CLS) for PE4, PE5, PE8, PE12 and PE21 were inverted U-shaped, whereas the dose-response curve for PE6 was J-shaped (Figure S1–S3). Such nonlinear and biphasic dose-response curves denote a hormetic kind of stress response, in which 1) lower (hormetic) concentrations of a chemical compound increase the survival of a cell or an organism by stimulating biological processes that allow to maintain cellular or organismal stress at a level which is below a threshold of toxicity; while 2) higher concentrations of this chemical compound decrease the survival of a cell or an organism by creating stress which exceeds such threshold [25, 45–48].

### Each of the six lifespan-extending PEs alters the age-related chronology of longevity-defining traits of mitochondrial functionality

We hypothesized that PE4, PE5, PE6, PE8, PE12 and PE21 slow yeast chronological aging by influencing certain cellular processes. We sought to identify these longevity-defining processes. Certain aspects of mitochondrial functionality (such as mitochondrial respiration, mitochondrial membrane potential and mitochondrial reactive oxygen species [ROS] homeostasis) are known to define the rate of chronological aging in yeast [6, 11, 23, 34, 52, 55–67]. We therefore assessed how PE4, PE5, PE6, PE8, PE12 and PE21 impact these longevity-defining processes in chronologically aging yeast cultures under non-CR conditions on 2% glucose.

**Figure 5:**
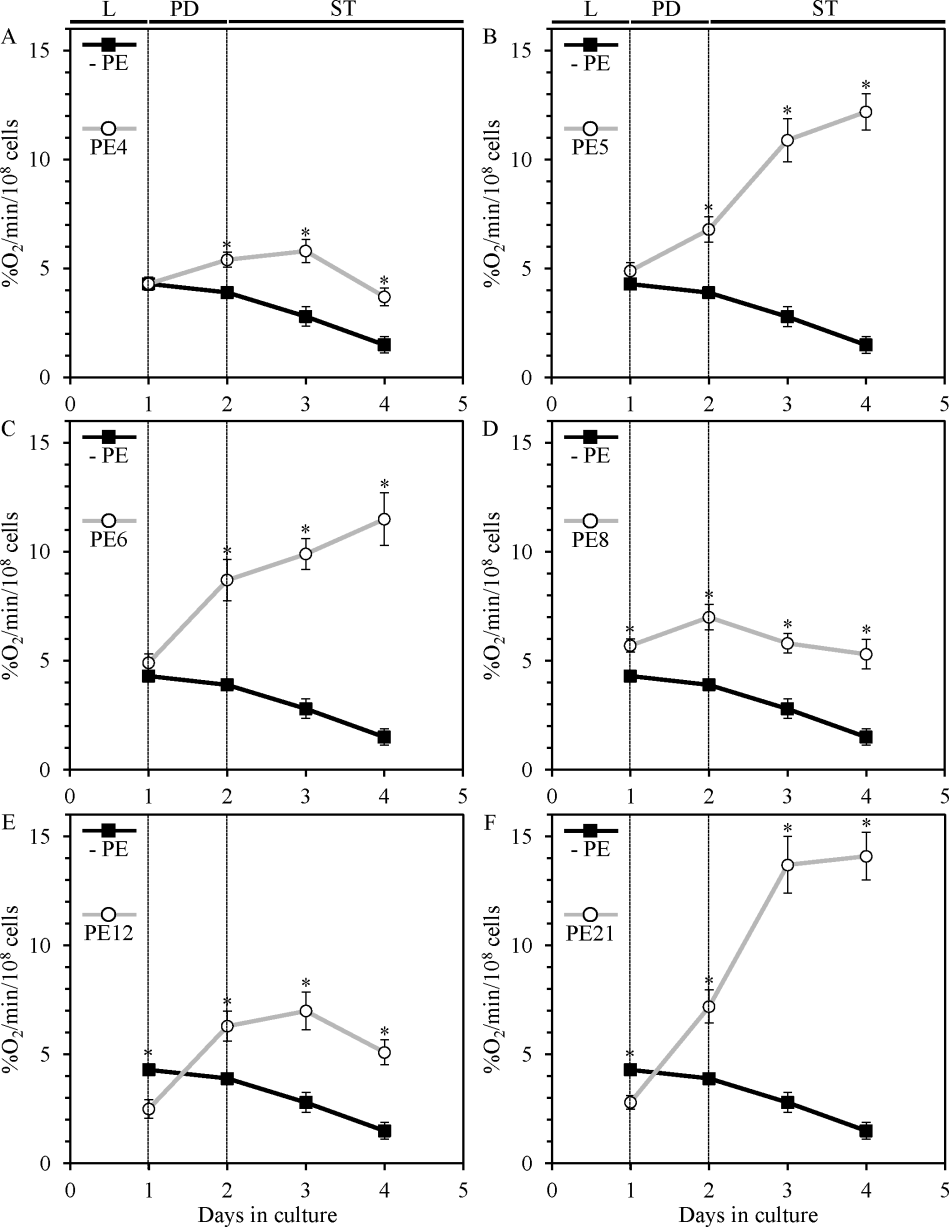
PE4, PE5, PE6, PE8, PE12 and PE21 alter the age-related chronology of mitochondrial oxygen consumption by yeast grown under non-CR conditions WT cells were grown in the synthetic minimal YNB medium initially containing 2% glucose, in the presence of a PE or in its absence. A polarographic assay was used to measure oxygen uptake by live yeast cells, as described in Materials and methods. Age-dependent changes in the rate of mitochondrial oxygen consumption by chronologically aging WT strain cultured under non-CR conditions on 2% glucose with or without 0.5% PE4 (**A**), 0.5% PE5 (**B**), 1% PE6 (**C**), 0.3% PE8 (**D**), 0.1% PE12 (**E**) or 0.1% PE21 (**F**) are shown; data are presented as means ± SEM (*n* = 7–9; *p < 0.05; the *p* values for comparing the means of two groups were calculated with the help of the GraphPad Prism statistics software using an unpaired two-tailed *t* test). Abbreviations: Logarithmic (L), post-diauxic (PD) or stationary (ST) growth phase.

We found that each of the six lifespan-extending PEs stimulates coupled mitochondrial respiration, which was monitored by measuring the rate of oxygen consumption by yeast cells. PE4, PE8 and PE12 decreased the extent to which such respiration declined in ST-phase cultures (Figure 5A, 5D and 5E), whereas PE5, PE6 and PE21 considerably increased the rate of mitochondrial respiration in yeast during PD and ST growth phases (Figure 5B, 5C and 5F).

**Figure 6:**
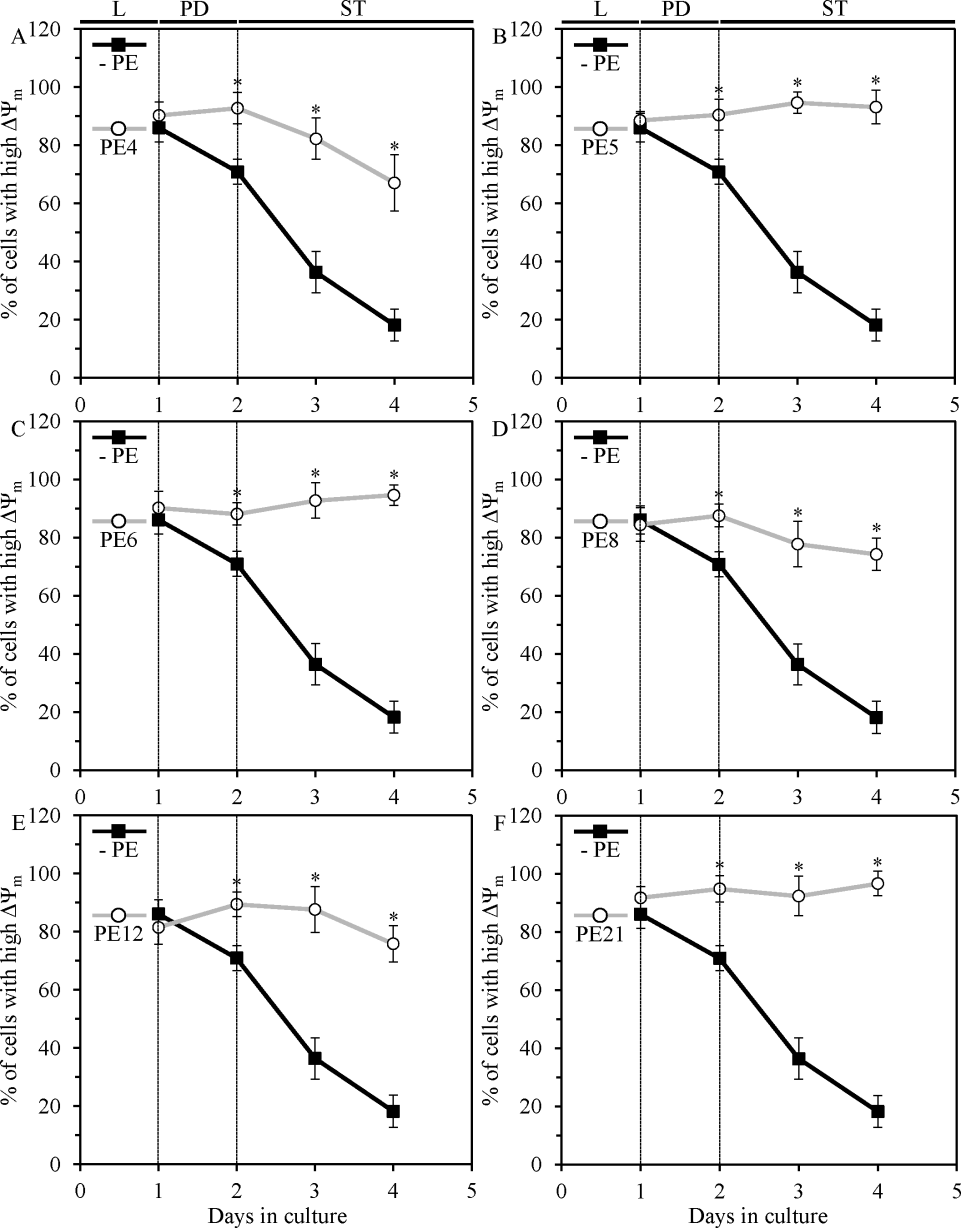
PE4, PE5, PE6, PE8, PE12 and PE21 sustain healthy populations of functional mitochondria that exhibit high mitochondrial membrane potential (ΔΨ_m_) in chronologically aging yeast grown under non-CR conditions WT cells were grown in the synthetic minimal YNB medium initially containing 2% glucose, in the presence of a PE or in its absence. ΔΨ_m_ was measured in live yeast by fluorescence microscopy of Rhodamine 123 staining, as described in Materials and methods. Age-dependent changes in the percentage of WT cells displaying high ΔΨ_m_ in chronologically aging yeast cultures under non-CR conditions on 2% glucose with or without 0.5% PE4 (**A**), 0.5% PE5 (**B**), 1% PE6 (**C**), 0.3% PE8 (**D**), 0.1% PE12 (**E**) or 0.1% PE21 (**F**) are shown; data are presented as means ± SEM (*n* = 3–4; *p < 0.05; the *p* values for comparing the means of two groups were calculated with the help of the GraphPad Prism statistics software using an unpaired two-tailed *t* test). Abbreviations: Logarithmic (L), post-diauxic (PD) or stationary (ST) growth phase.

We also found that each of the six lifespan-extending PEs sustains healthy populations of functional mitochondria that exhibit high mitochondrial membrane potential (ΔΨ_m_). PE4, PE8 and PE12 substantially reduced the extent to which ΔΨ_m_ declined during PD and ST growth phases (Figure 6A, 6D and 6E; Figure S8 and S9), whereas PE5, PE6 and PE21 completely prevented such decline (Figure 6B, 6C and 6F; Figure S8 and S9).

**Figure 7:**
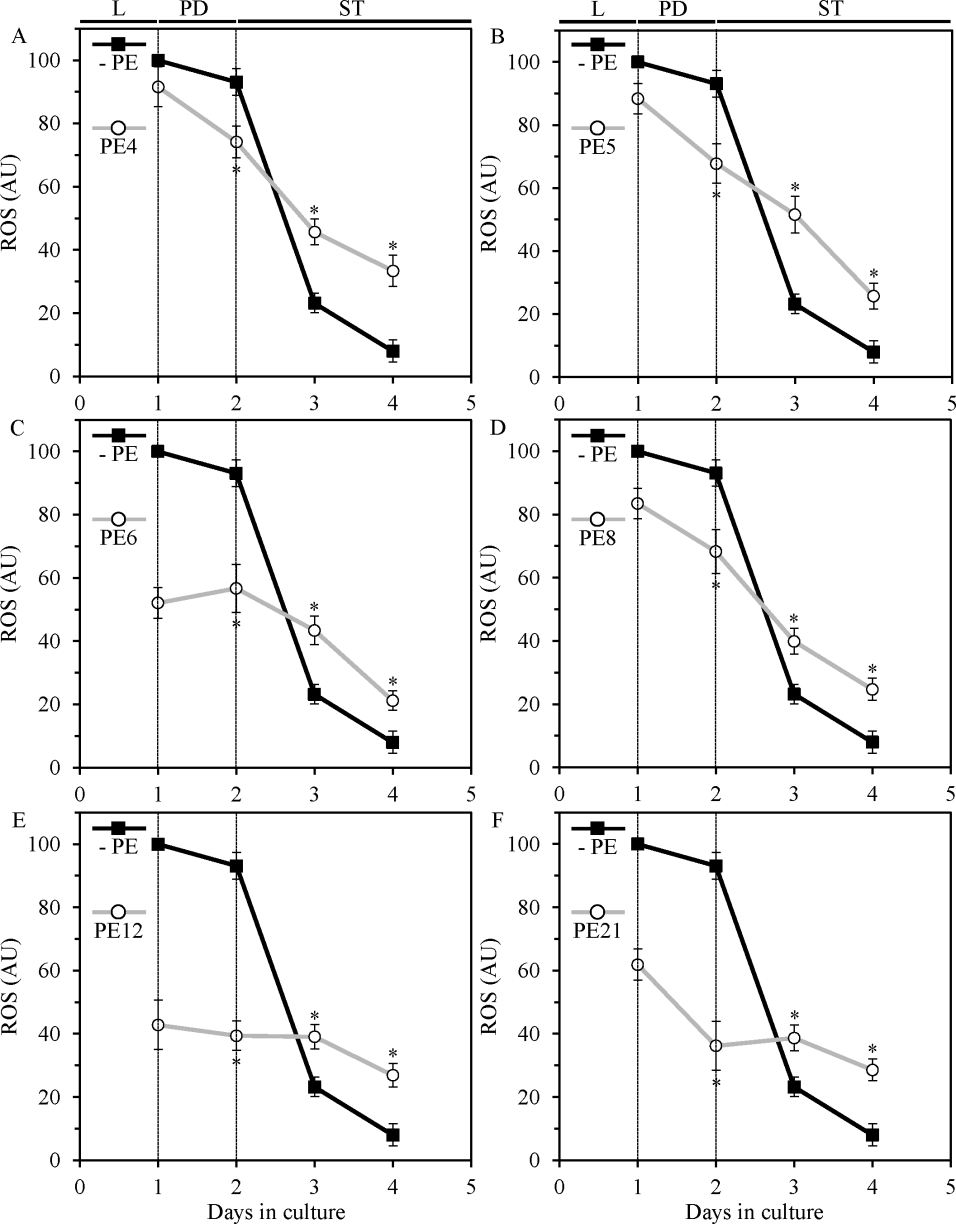
In yeast grown under non-CR conditions, PE4, PE5, PE6, PE8, PE12 and PE21 alter the patterns of age-related changes in intracellular reactive oxygen species (ROS) known to be generated mainly as by-products of mitochondrial respiration WT cells were grown in the synthetic minimal YNB medium initially containing 2% glucose, in the presence of a PE or in its absence. The intracellular concentrations of ROS were measured in live yeast by fluorescence microscopy of dihydrorhodamine 123 staining, as described in Materials and methods. Age-dependent changes in ROS concentrations within chronologically aging WT cells cultured under non-CR conditions on 2% glucose with or without 0.5% PE4 (**A**), 0.5% PE5 (**B**), 1% PE6 (**C**), 0.3% PE8 (**D**), 0.1% PE12 (**E**) or 0.1% PE21 (**F**) are shown; data are presented as means ± SEM (*n* = 3–4; *p < 0.05; the *p* values for comparing the means of two groups were calculated with the help of the GraphPad Prism statistics software using an unpaired two-tailed *t* test). Abbreviations: Logarithmic (L), post-diauxic (PD) or stationary (ST) growth phase.

PE4, PE5, PE6, PE8, PE12 and PE21 also caused significant changes in the age-related chronology of intracellular ROS, which in yeast and other organisms are known to be formed mainly as by-products of mitochondrial respiration [68, 69]. Each of these PEs decreased the extent to which the intracellular concentration of mitochondrially generated ROS declined during PD and ST growth phases (Figure 7A–7F). On days 3 and 4 of culturing, ROS concentrations in yeast grown with PE4, PE5, PE6, PE8, PE12 or PE21 exceeded those in yeast grown without it (Figure 7A–7F).

### The six lifespan-extending PEs differently influence the extent of an age-related oxidative damage to cellular proteins, membrane lipids, mitochondrial and nuclear genomes

A body of evidence supports the following view on the relationships between cellular ROS, oxidative molecular damage and aging in organisms across phyla: 1) if cellular concentrations of ROS exceed a threshold of toxicity, ROS cause oxidative damage to proteins, lipids and DNA; 2) oxidative damage to each kind of these macromolecules accumulates with age; and 3) cumulative oxidative damage to the different kinds of macromolecules is one of the major causes of aging [53, 54, 69–75]. We therefore examined how PE4, PE5, PE6, PE8, PE12 and PE21 impact the extent of oxidative damage to proteins, lipids and DNA in chronologically aging yeast cultured under non-CR conditions on 2% glucose.

**Figure 8:**
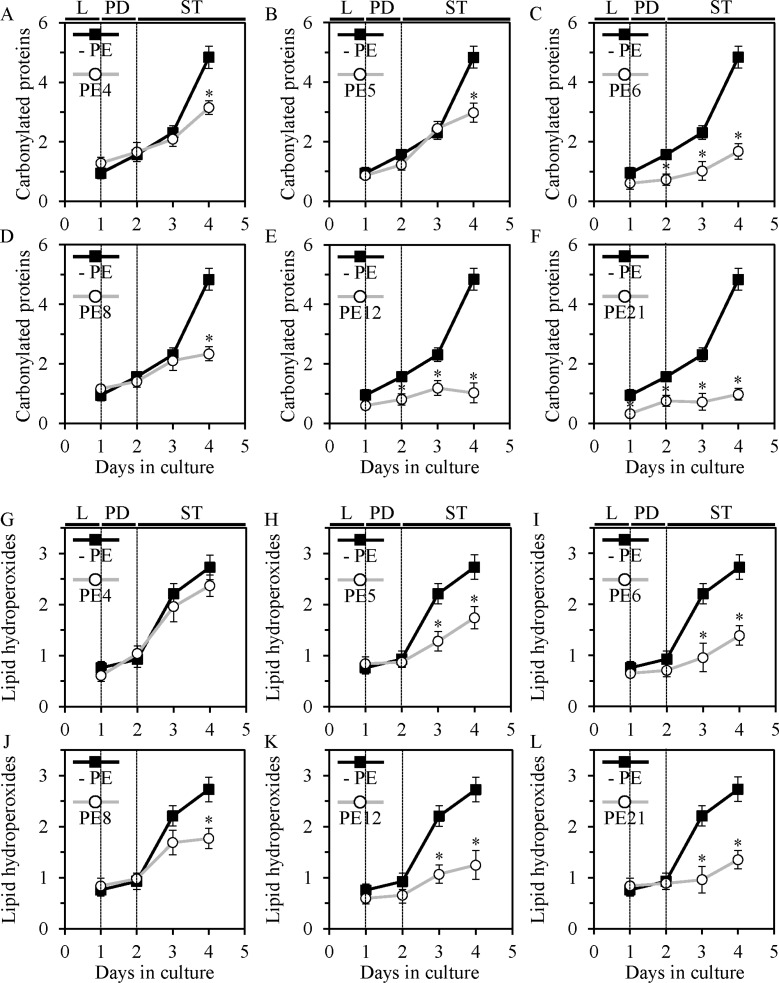
PE4, PE5, PE6, PE8, PE12 and PE21 delay an age-dependent rise in the extent of oxidative damage to cellular proteins in chronologically aging yeast grown under non-CR conditions. PE5, PE6, PE8, PE12 and PE21, but not PE4, have similar effects on the extent of oxidative damage to membrane lipids WT cells were grown in the synthetic minimal YNB medium initially containing 2% glucose, in the presence of a PE or in its absence. Carbonylated cellular proteins (**A–F**) and oxidatively damaged membrane lipids (**G–L**) were determined as described in Materials and methods. Age-dependent changes in the concentrations of these oxidatively damaged macromolecules within chronologically aging WT cells cultured under non-CR conditions on 2% glucose with or without 0.5% PE4 (**A** and **G**), 0.5% PE5 (**B** and **H**), 1% PE6 (**C** and **I**), 0.3% PE8 (**D** and **J**), 0.1% PE12 (**E** and **K**) or 0.1% PE21 (**F** and **L**) are shown; data are presented as means ± SEM (*n* = 2–4; *p < 0.05; the *p* values for comparing the means of two groups were calculated with the help of the GraphPad Prism statistics software using an unpaired two-tailed *t* test). Abbreviations: Logarithmic (L), post-diauxic (PD) or stationary (ST) growth phase.

We found that each of the six lifespan-extending PEs delays an age-dependent rise in the extent of oxidative damage to cellular proteins. PE6, PE12 and PE21 reduced oxidative carbonylation of proteins in yeast cells progressing through the entire ST phase (Figure 8C, 8E and 8F). PE4, PE5 and PE8 elicited such inhibitory effect on oxidative protein damage only later in ST phase, on day 4 of culturing (Figure 8A, 8B and 8D).

Furthermore, PE5, PE6, PE8, PE12 and PE21 (but not PE4) caused a significant reduction in the levels of oxidatively damaged membrane lipids; such reduction was observed late in ST phase, on days 3 and/or 4 of culturing (Figure 8H–8L).

**Figure 9:**
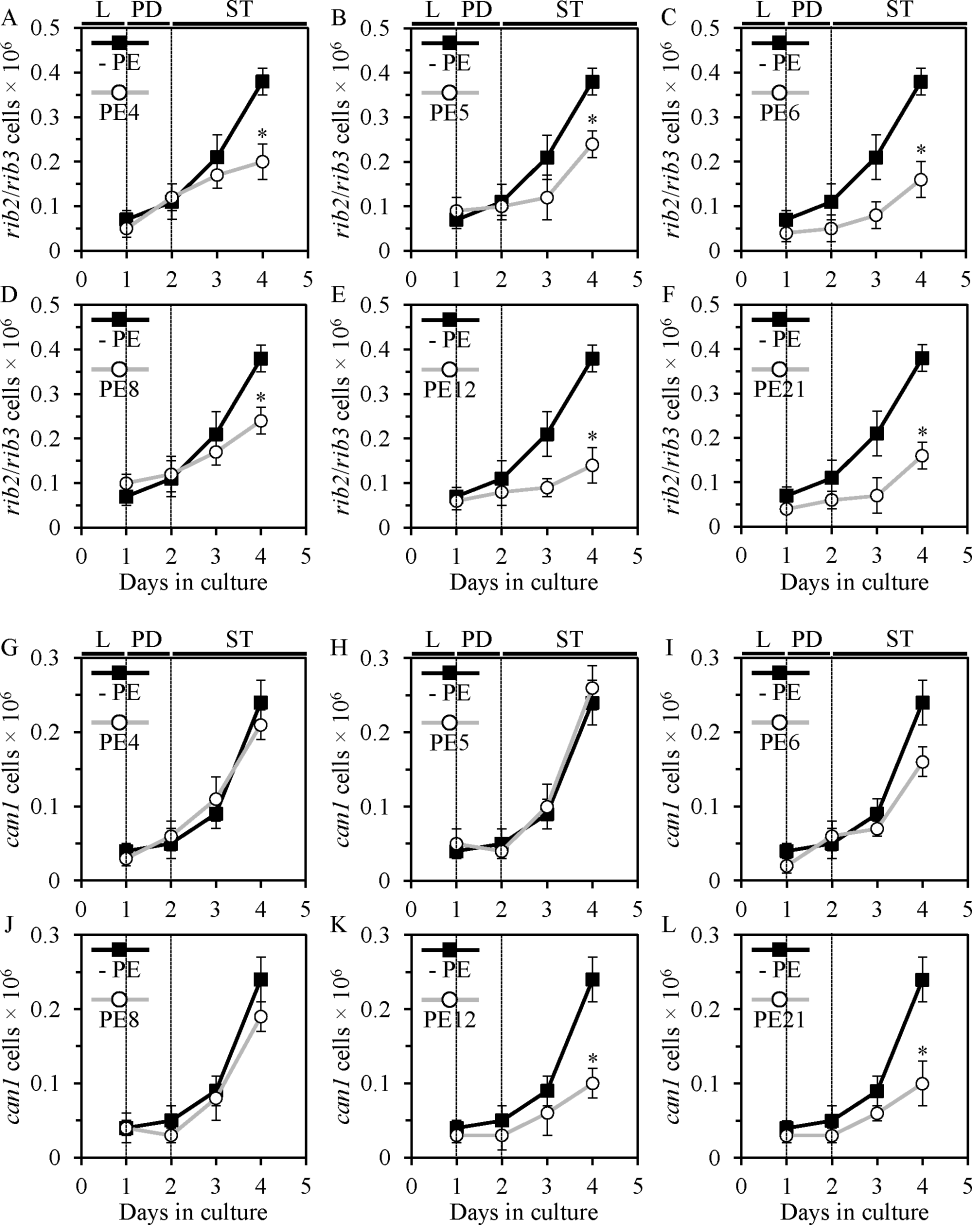
PE4, PE5, PE6, PE8, PE12 and PE21 slow down an age-dependent rise in the frequency of spontaneous point mutations in the *rib2* and *rib3* loci of mitochondrial DNA (mtDNA) in chronologically aging yeast grown under non-CR conditions. PE12 and PE21, but not PE4, PE5, PE6 or PE8, have similar effects on the frequency of spontaneous point mutations in the *CAN1* gene of nuclear DNA (nDNA) WT cells were grown in the synthetic minimal YNB medium initially containing 2% glucose, in the presence of a PE or in its absence. The frequency of spontaneous point mutations in the *rib2* and *rib3* loci of mtDNA (**A–F**), as well as the frequency of spontaneous point mutations in the *CAN1* gene of nDNA (**G–L**), were measured as described in Materials and methods. Age-dependent changes in the frequencies of these mtDNA and nDNA mutations in chronologically aging WT cells cultured under non-CR conditions on 2% glucose with or without 0.5% PE4 (**A** and **G**), 0.5% PE5 (**B** and **H**), 1% PE6 (**C** and **I**), 0.3% PE8 (**D** and **J**), 0.1% PE12 (**E** and **K**) or 0.1% PE21 (**F** and **L**) are shown; data are presented as means ± SEM (*n* = 3–5; *p < 0.05; the *p* values for comparing the means of two groups were calculated with the help of the GraphPad Prism statistics software using an unpaired two-tailed *t* test). Abbreviations: Logarithmic (L), post-diauxic (PD) or stationary (ST) growth phase.

Moreover, PE4, PE5, PE6, PE8, PE12 and PE21 decreased the frequencies of spontaneous point mutations in the *RIB2* and *RIB3* genes of mitochondrial DNA (mtDNA) (Figure 9A–9F) - likely due to a reduced extent of oxidative damage to mtDNA in yeast cells exposed to any of these PEs. Such inhibitory effects of the six lifespan-extending PEs on oxidative damage to mtDNA was observed late in ST phase, on day 4 of culturing.

We also revealed that PE12 and PE21 (but not PE4, PE5, PE6 or PE8) caused a significant reduction in the frequencies of spontaneous point mutations in the *CAN1* gene of nuclear DNA (nDNA) (Figure 9K and 9L) - possibly due to a decreased degree of oxidative damage to nDNA in yeast cells grown in the presence of PE12 or PE21. Such inhibitory effects of PE12 or PE21 on oxidative damage to nDNA was also seen late in ST phase, on day 4 of culturing.

### The six lifespan-extending PEs differently influence the resistance of chronologically aging yeast to chronic oxidative and thermal stresses

A body of evidence implies that the development of resistance to chronic (long-term) oxidative and/or thermal stresses can extend longevity in organisms across phyla, including yeast [6, 9, 10, 11, 34, 46–48, 68, 76–80]. We therefore assessed how PE4, PE5, PE6, PE8, PE12 and PE21 influence the abilities of chronologically aging yeast cultured under non-CR conditions to resist chronic oxidative and thermal stresses.

**Figure 10:**
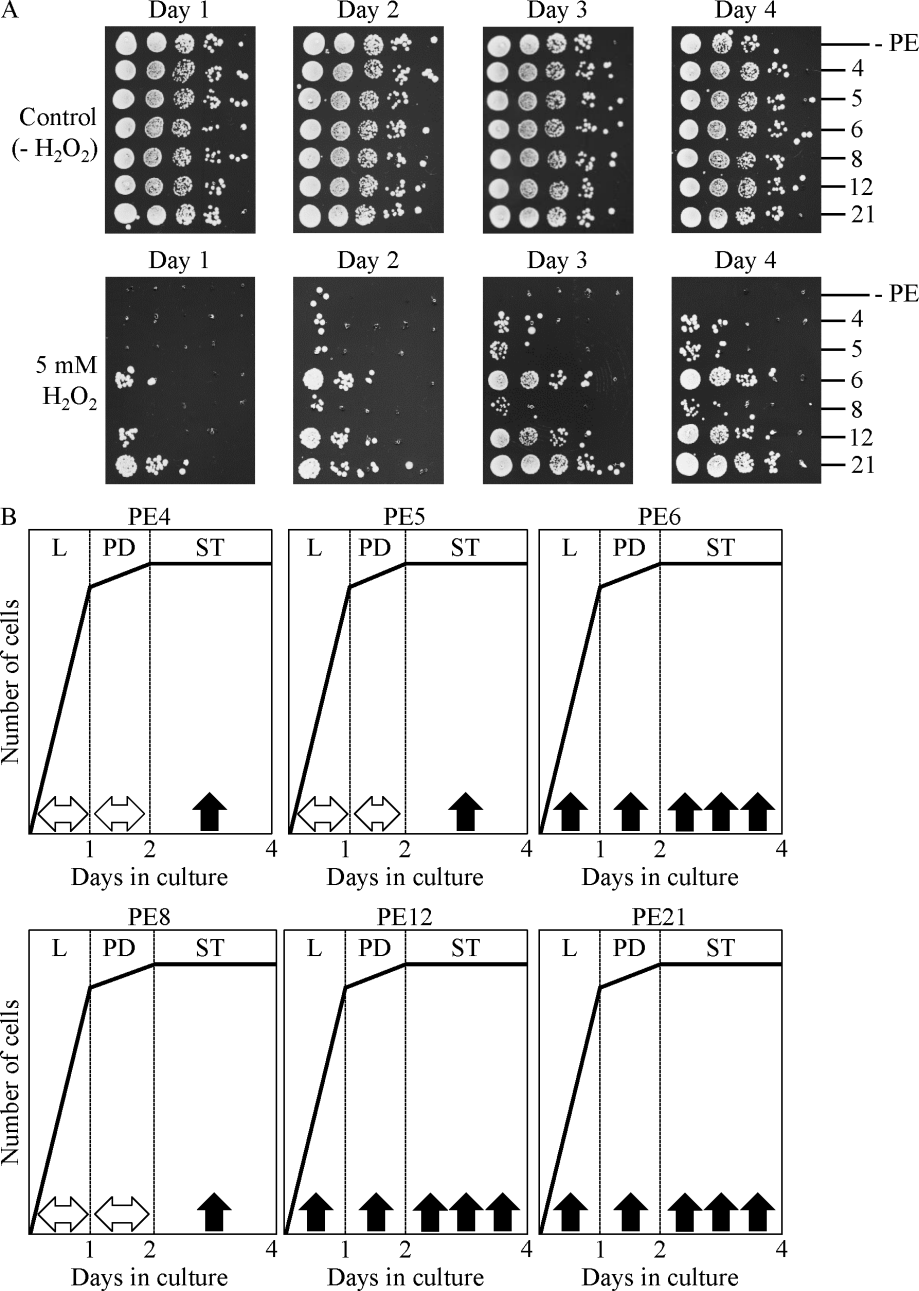
PE4, PE5, PE6, PE8, PE12 and PE21 enhance the ability of chronologically aging yeast grown under non-CR conditions to resist chronic oxidative stress WT cells were grown in the synthetic minimal YNB medium initially containing 2% glucose, in the presence of a PE or in its absence. (**A**) Spot assays for monitoring oxidative stress resistance were performed as described in Materials and methods. Serial 10-fold dilutions of cells recovered at different days of culturing were spotted on plates with solid YEP medium containing 2% glucose as carbon source, with or without 5 mM hydrogen peroxide. All pictures were taken after a 3-d incubation at 30°C. (**B**) A model for how 0.5% PE4, 0.5% PE5, 1% PE6, 0.3% PE8, 0.1% PE12 and 0.1% PE21 influence the resistance of yeast to chronic oxidative stress during logarithmic (L), post-diauxic (PD) or stationary (ST) phases of growth. 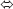 or 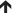 Denote unaltered or enhanced, respectively, cell resistance to chronic oxidative stress during a particular phase of growth. Abbreviations: Logarithmic (L), post-diauxic (PD) or stationary (ST) growth phase.

Chronic oxidative stress was administered by recovering yeast cells progressing through L, PD or ST phases of growth/culturing in liquid YNB medium initially containing 2% glucose, spotting these cells on solid YEP medium with 2% glucose and 5 mM hydrogen peroxide, and incubating them for 3 days. We found that PE6, PE12 and PE21 significantly increase cell resistance to chronic oxidative stress in yeast cultures progressing through L, PD and ST phases (Figure 10A and 10B). PE4, PE5 and PE8 enhanced the ability of cells to resist chronic oxidative stress only in yeast cultures progressing through ST phase, but did not alter such ability during L and PD phases (Figure 10A and 10B).

**Figure 11:**
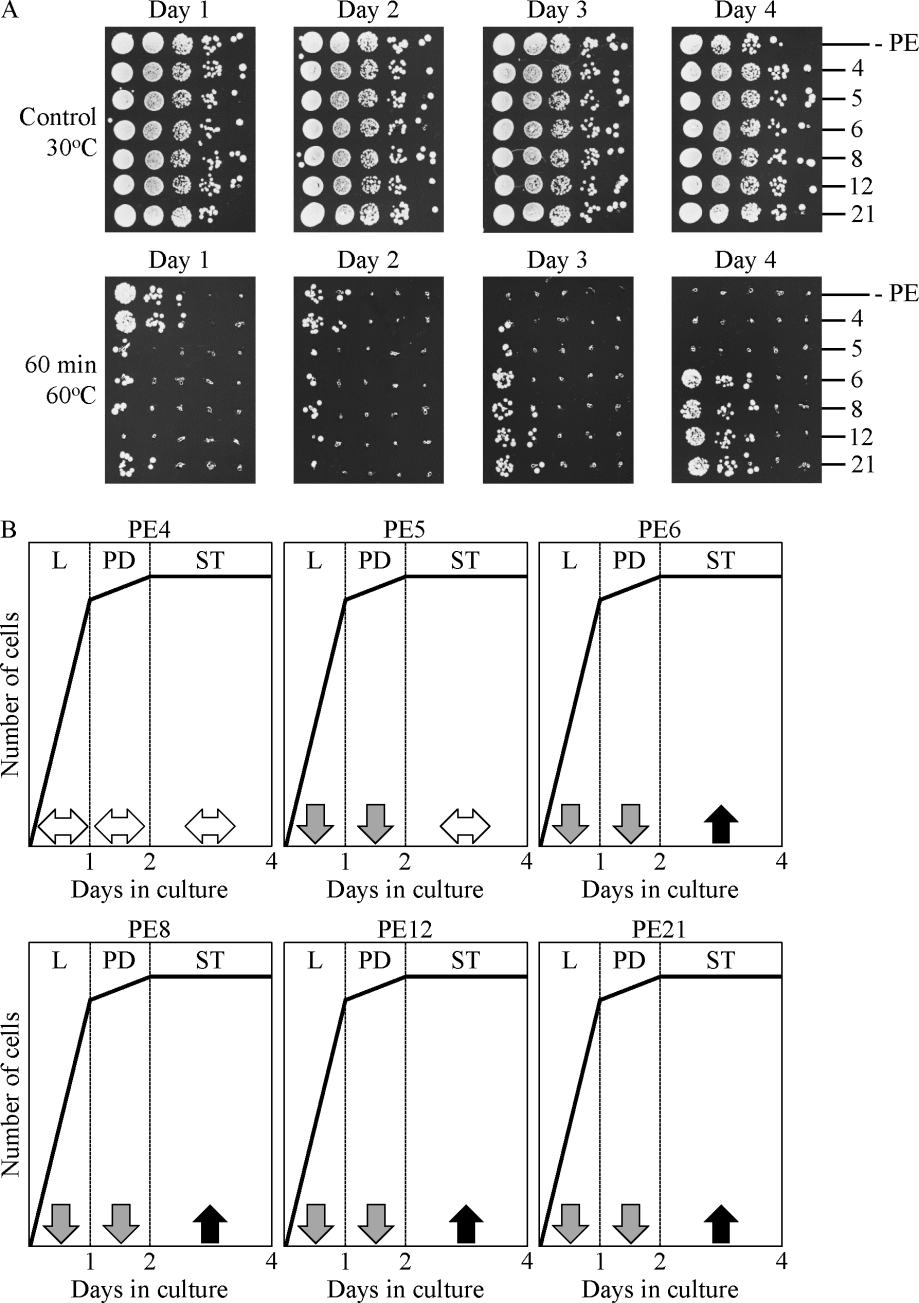
PE4, PE5, PE6, PE8, PE12 and PE21 exhibit different effects on the ability of chronologically aging yeast grown under non-CR conditions to resist chronic thermal stress WT cells were grown in the synthetic minimal YNB medium initially containing 2% glucose, in the presence of a PE or in its absence. (**A**) Spot assays for monitoring thermal stress resistance were performed as described in Materials and methods. Serial 10-fold dilutions of cells recovered at different days of culturing were spotted on plates with solid YEP medium containing 2% glucose as carbon source. Plates were initially incubated at 30°C (control) or 60°C for 60 min, and were then transferred to 30°C. All pictures were taken after a 3-d incubation at 30°C. (**B**) A model for how 0.5% PE4, 0.5% PE5, 1% PE6, 0.3% PE8, 0.1% PE12 and 0.1% PE21 influence the resistance of yeast to chronic thermal stress during logarithmic (L), post-diauxic (PD) or stationary (ST) phases of growth. 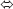, 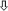 or 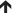 Denote unaltered, reduced or enhanced, respectively, cell resistance to chronic thermal stress during a particular phase of growth. Abbreviations: Logarithmic (L), post-diauxic (PD) or stationary (ST) growth phase.

Chronic thermal stress was administered by recovering yeast cells progressing through L, PD or ST phases of growth/culturing in liquid YNB medium initially containing 2% glucose, spotting these cells on solid YEP medium with 2% glucose and incubating at 60°C for 60 min, and then transferring plates with these cells to 30°C and incubating at this temperature for 3 days. We found that PE6, PE8, PE12 and PE21 increase cell resistance to chronic thermal stress only in yeast cultures progressing through ST phase (Figure 11A and 11B). In contrast, each of these four lifespan-extending PEs weakened the ability of cells to resist chronic thermal stress during L and PD phases (Figure 11A and 11B). Furthermore, neither PE4 nor PE5 altered cell resistance to chronic thermal stress in yeast cultures progressing through ST phase (Figure 11A and 11B). PE4 did not affect the ability of cells to resist chronic thermal stress also during L and PD phases, whereas PE5 reduced such ability in yeast cultures progressing through these two phases (Figure 11A and 11B).

### Each of the six lifespan-extending PEs causes rapid degradation of neutral lipids deposited in lipid droplets

Triacylglycerols and steryl esters are uncharged (and therefore are called “neutral” or “nonpolar”) classes of lipids that can be found in cells of all eukaryotic organisms [81–83]. After being initially synthesized in the endoplasmic reticulum and then deposited in lipid droplets (LDs), these two highly hydrophobic lipids can undergo lipolytic degradation to provide substrates for the synthesis of phospholipids and sphingolipids [82, 84–87]. Emergent evidence supports the view that the biosynthesis, storage and lipolysis of neutral lipids are longevity assurance processes; importantly, it has been shown that these processes can be controlled by certain dietary and pharmacological interventions known to delay aging in various eukaryotes, including yeast [6, 23, 34, 87–108]. We therefore used live-cell fluorescence microscopy to examine how PE4, PE5, PE6, PE8, PE12 and PE21 influence the age-related dynamics of changes in the intracellular concentration of neutral lipids confined to LDs in chronologically aging yeast grown under non-CR conditions.

**Figure 12:**
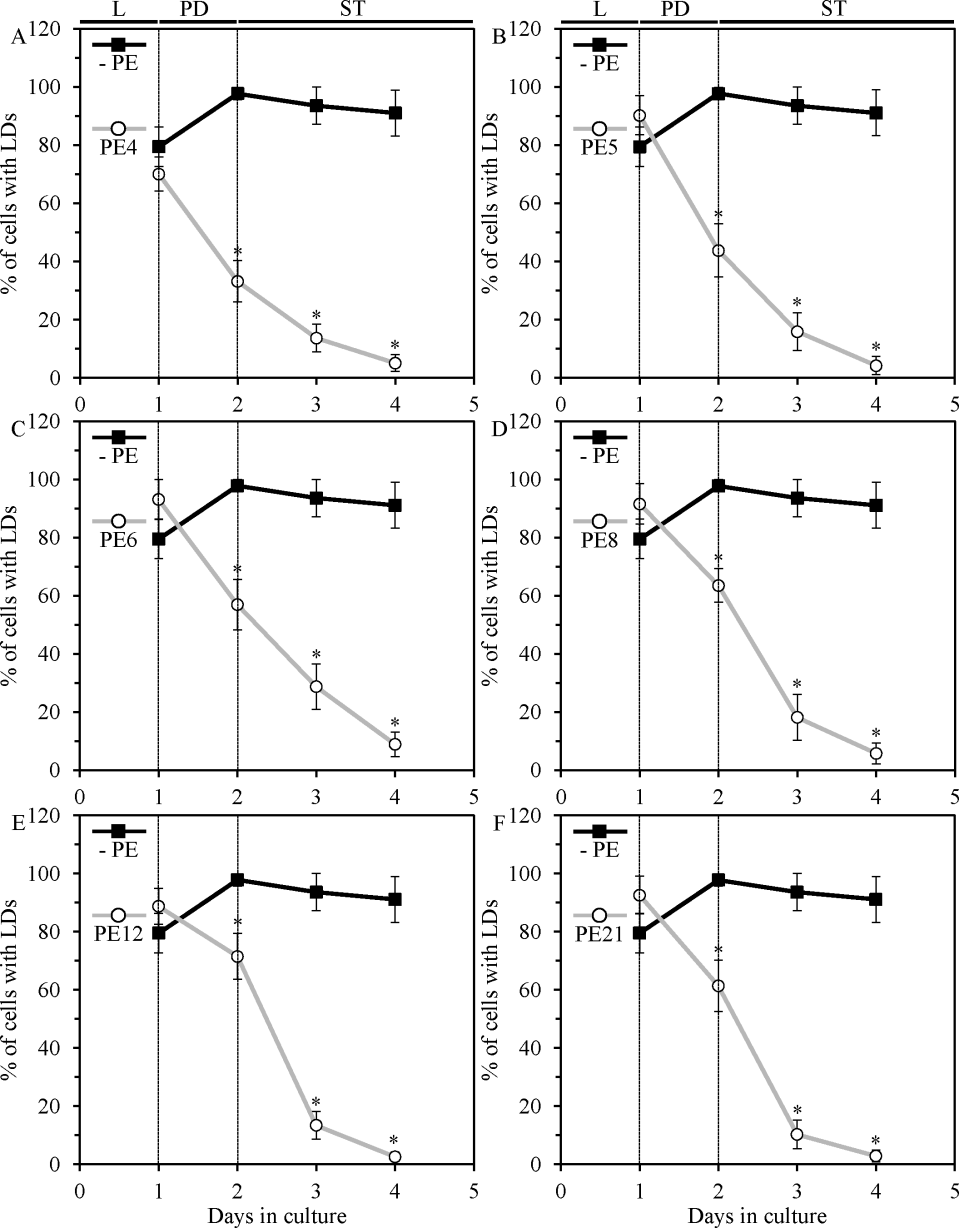
PE4, PE5, PE6, PE8, PE12 and PE21 induce rapid consumption of neutral lipids deposited in lipid droplets (LDs) of chronologically aging yeast grown under non-CR conditions WT cells were grown in the synthetic minimal YNB medium initially containing 2% glucose, in the presence of a PE or in its absence. Neutral lipids deposited in LDs were measured in live yeast by fluorescence microscopy of BODIPY 493/503 staining, as described in Materials and methods. Age-dependent changes in the percentage of WT cells exhibiting LDs in chronologically aging yeast cultures under non-CR conditions on 2% glucose with or without 0.5% PE4 (**A**), 0.5% PE5 (**B**), 1% PE6 (**C**), 0.3% PE8 (**D**), 0.1% PE12 (**E**) or 0.1% PE21 (**F**) are shown; data are presented as means ± SEM (*n* = 3–4; *p < 0.05; the *p* values for comparing the means of two groups were calculated with the help of the GraphPad Prism statistics software using an unpaired two-tailed *t* test). Abbreviations: Logarithmic (L), post-diauxic (PD) or stationary (ST) growth phase.

We found that each of the six lifespan-extending PEs elicits rapid age-related decline in the number of yeast cells exhibiting LDs (Figures 12A–12F; Figure S10 and S11). In contrast, no significant changes in the number of cells with LDs were seen in yeast progressing through L, PD and ST phases of culturing in medium without a PE (Figure 12A–12F; Figures S10 and S11). These findings demonstrate that in chronologically aging yeast grown under non-CR conditions, each of the six lifespan-extending PEs causes rapid lipolytic degradation of neutral lipids stored in LDs.

## DISCUSSION

**Figure 13:**
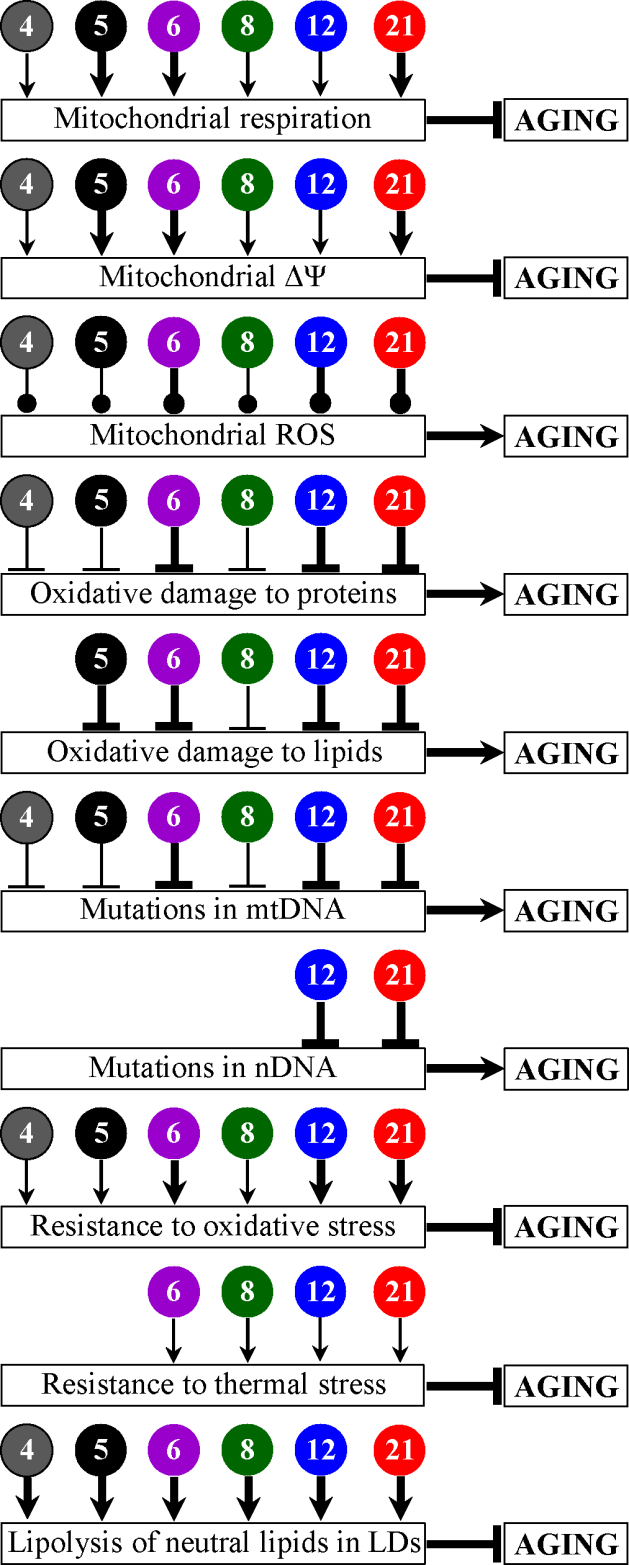
PE4, PE5, PE6, PE8, PE12 and PE21 delay yeast chronological aging and have different effects on several longevity-defining cellular processes Arrows pointing at boxes with the terms of longevity-defining cellular processes denote activation of these processes, T bars denote inhibition of these processes, whereas lines with filled circles denote change in the age-related chronology of intracellular ROS. The thickness of such arrows, T bars and lines with filled circles correlates with the extent to which a PE activates, inhibits or alters the age-related chronology (respectively) of a particular longevity-defining cellular process. Arrows and T bars pointing at boxes with the term “AGING” denote acceleration or deceleration (respectively) of yeast chronological aging.

In this study, we performed a screen for PEs capable of extending longevity of the chronologically aging yeast *S. cerevisiae*. Our screen revealed six PEs (which we call PE4, PE5, PE6, PE8, PE12 and PE21) that can significantly increase yeast CLS. We demonstrated that each of these PEs is a geroprotector which delays the onset and slows the progression of yeast chronological aging by eliciting a hormetic stress response. We provided evidence that each of these geroprotective PEs has different effects on cellular processes known to define longevity in organisms across phyla. Such effects include the following: 1) amplified mitochondrial respiration and membrane potential; 2) increased or decreased concentrations of ROS; 3) reduced oxidative damage to cellular proteins, membrane lipids, and mitochondrial and nuclear genomes; 4) enhanced cell resistance to oxidative and thermal stresses; and 5) accelerated degradation of neutral lipids deposited in LDs (Figure 13). These findings provide important new insights into mechanisms through which some chemical compounds of plant origin can slow biological aging.

### Each of the six longevity-extending PEs increases lifespan more efficiently than any lifespan-prolonging chemical compound currently known

Our findings imply that the efficiency of longevity extension by PE4, PE5, PE6, PE8, PE12 or PE21 greatly exceeds that for any of the 42 chemical compounds known to increase lifespan in yeasts, filamentous fungi, nematodes, fruit flies, daphnias, mosquitoes, honey bees, fishes, mammals and cultured human cells (Table S1). Indeed, under non-CR conditions these longevity-extending PEs increase the mean and maximum CLS of yeast by 145%–475% and 80%–369%, respectively (Figure 3A and 3B; Table S1); the corresponding rows in Table S1 are highlighted in yellow. In contrast, any of the 42 currently known lifespan-extending chemical compounds has been shown to extend cellular and/or organismal lifespan in evolutionarily distant eukaryotes much less efficiently, within the 5% to 75% range (Table S1) [references 1–27, 30–57, 59–61 for Table S1]. Only two chemical compounds, spermidine under non-CR conditions and lithocholic acid under CR-conditions, have been reported to exhibit the lifespan-extending efficiencies that are comparable to those for PE4, PE5, PE6, PE8 and PE12 (Table S1) [references 28, 29, 58 for Table S1]. Specifically, both these pharmacological interventions were demonstrated to increase the RLS and/or CLS of yeast and human peripheral blood mononuclear cells by 83%–200%; the corresponding rows in Table S1 are highlighted in green. Of note, PE21 appears to be the most potent longevity-extending pharmacological intervention presently known. It increases the mean and maximum CLS of yeast by 475% and 369%, respectively (Figure 3A and 3B; Table S1).

### Future perspectives

In the future, it would be important to further explore the following key aspects of the mechanisms through which each of the six longevity-extending PEs slows biological aging.

First, it is intriguing to identify the individual chemical compounds responsible for the ability of each of these PEs to delay the onset and decrease the rate of yeast chronological aging. Such identification is already underway in our laboratory; of note, it is conceivable that only some combinations of certain chemicals composing these PEs (but not individual chemical compounds per se) can be responsible for their extremely high efficiencies as aging-delaying interventions.

Second, it is interesting to elucidate how genetic interventions that impair any of the few nutrient- and energy-sensing signaling pathways known to define longevity of chronologically aging yeast [6, 10, 11, 23] influence the extent to which each of the six longevity-extending PEs can slow aging. These studies may allow to identify protein components of the longevity-defining signaling pathways that are targeted by each of the PEs. These studies may also reveal that certain combinations of these PEs and genetically impaired components of pro-aging signaling pathways exhibit additive or synergistic effects on the efficiencies of lifespan and healthspan extensions.

Third, it is important to investigate how various combinations of the six longevity-extending PEs with each other and with presently known aging-delaying chemical compounds alter the extent of CLS extension in yeast. These studies may identify such combinations of various pharmacological interventions that impose substantial additive or synergistic effects on the efficiencies with which organismal lifespan and healthspan can be prolonged.

Fourth, our ongoing studies indicate that the six longevity-extending PEs also extend longevities of other eukaryotic model organisms, delay the onset of age-related diseases and/or exhibit anti-tumor effects. In this regard, it needs to be mentioned that genetic, dietary and pharmacological interventions known to delay aging in yeast and other eukaryotes have been shown to selectively kill cultured human cancer cells and/or decrease the incidence of cancer [29, 88, 109–120]. The challenge for the future is to define mechanisms through which the six geroprotective PEs prolong healthy lifespan and decelerate tumorigenesis.

## MATERIALS AND METHODS

### Yeast strains, media and growth conditions

The wild-type strain *Saccharomyces cerevisiae* BY4742 (*MAT*a *his3D1 leu2D0 lys2D0 ura3D0*) from Thermo Scientific/Open Biosystems was grown in a synthetic minimal YNB medium (0.67% Yeast Nitrogen Base without amino acids) initially containing 2% or 0.5% glucose and supplemented with 20 mg/l histidine, 30 mg/l leucine, 30 mg/l lysine and 20 mg/l uracil. Cells were cultured at 30^°^C with rotational shaking at 200 rpm in Erlenmeyer flasks at a “flask volume/medium volume” ratio of 5:1.

### CLS assay

A sample of cells was taken from a culture at a certain day following cell inoculation and PE addition into the medium. A fraction of the sample was diluted in order to determine the total number of cells using a hemacytometer. Another fraction of the cell sample was diluted and serial dilutions of cells were plated in duplicate onto YEP (1% yeast extract, 2% peptone) plates containing 2% glucose as carbon source. After 2 d of incubation at 30^°^C, the number of colony forming units (CFU) per plate was counted. The number of CFU was defined as the number of viable cells in a sample. For each culture, the percentage of viable cells was calculated as follows: (number of viable cells per ml/total number of cells per ml) × 100. The percentage of viable cells in mid-logarithmic growth phase was set at 100%.

### A screen for PEs that can extend yeast CLS

CLS analysis in the presence of various PEs was performed as described above. A 20% stock solution of each PE in ethanol was made on the day of adding this PE to cell cultures. For each PE, the stock solution was added to growth medium with 2% glucose immediately following cell inoculation into the medium. The final concentration of each PE in the medium was 0.02%, 0.04%, 0.06%, 0.08%, 0.1%, 0.3%, 0.5% or 1.0%.

### Miscellaneous procedures

The age-specific mortality rate (q_x_) [37, 42], Gompertz slope or mortality rate coefficient (α) [41, 42], and mortality rate doubling time (MRDT) [41, 42] were calculated as previously described. Oxygen consumption assay for monitoring mitochondrial respiration [34], mitochondrial membrane potential measurement in live yeast [34], ROS measurement in live yeast [121], BODIPY 493/503 staining for examining neutral lipids deposited in LDs [122], fluorescence microscopy [34], quantitative assays for oxidatively damaged proteins and membrane lipids [64], measurements of the frequencies of spontaneous mutations in mitochondrial and nuclear DNA [123], and plating assays for the analysis of resistance to oxidative and thermal stresses [123] have been described elsewhere.

### Statistical analysis

Statistical analysis was performed using Microsoft Excel's (2010) Analysis ToolPack-VBA. All data are presented as mean ± SEM. The *p* values for comparing the means of two groups (using an unpaired two-tailed *t* test) and survival curves (using a two-tailed *t* test) were calculated with the help of the GraphPad Prism statistics software.

## SUPPLEMENTARY MATERIAL FIGURES AND TABLE


