# Supplementary material for: Discovery of plant extracts that greatly delay yeast chronological aging and have different effects on longevity-defining cellular processes
Source: Oncotarget. 2016 Feb 24;7(13):16542–66. doi: 10.18632/oncotarget.7665 (PMC4941334; doi:10.18632/oncotarget.7665)
Supplement: Supplementary file 1 [file oncotarget-07-16542-s001.pdf]

Discovery of plant extracts that greatly delay yeast chronological aging and have different effects on longevity-defining cellular processes

Supplementary Materials

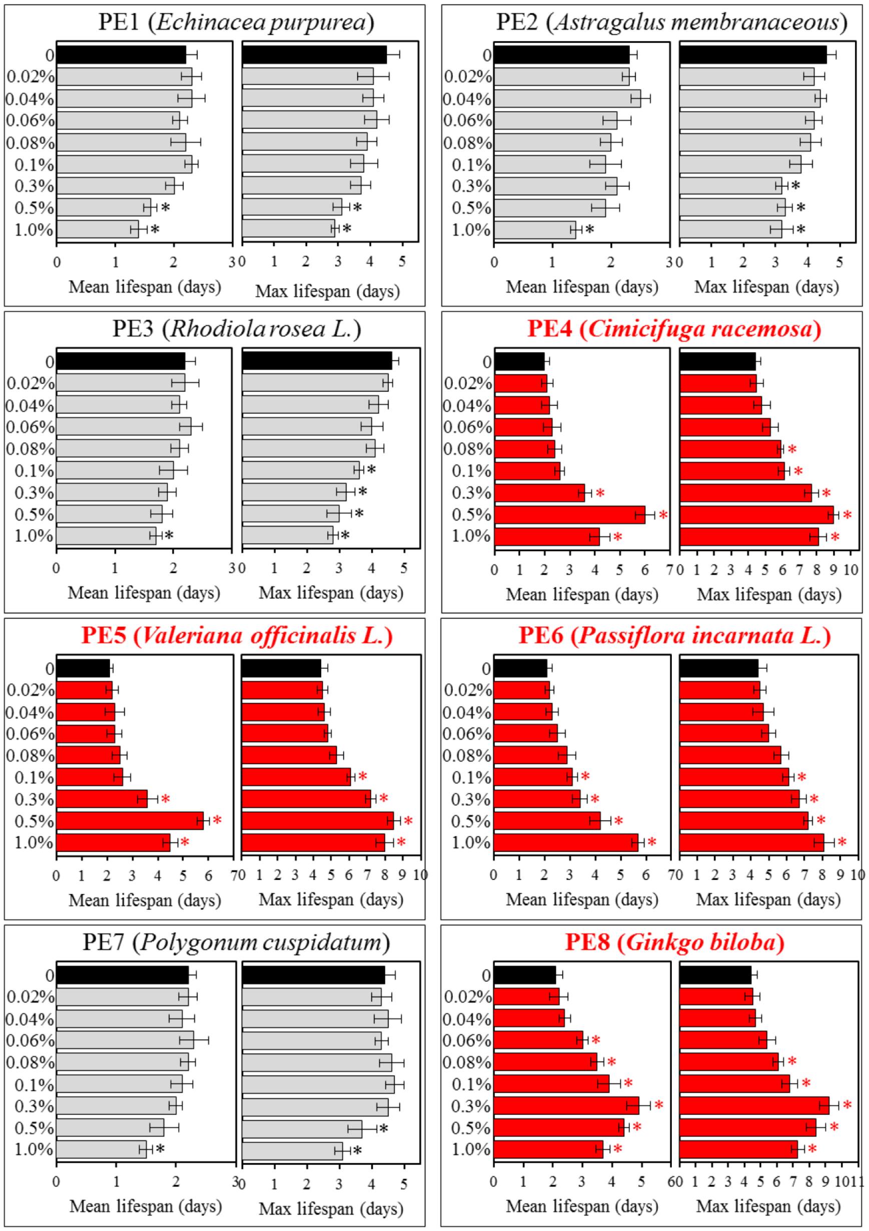

Supplementary Figure S1: PE4, PE5, PE6 and PE8, but not PE1, PE2, PE3 or PE7, extend the CLS of WT yeast grown under non-CR conditions. WT cells were grown in the synthetic minimal YNB medium initially containing 2% glucose (non-CR conditions), in the presence of a PE or in its absence. The mean and maximum lifespans of chronologically aging WT strain cultured under non-CR conditions without a PE or with a PE added at various concentrations are shown; data are presented as means ± SEM (n = 6–21; $*p < 0.05$; the p values for comparing the means of two groups were calculated with the help of the GraphPad Prism statistics software using an unpaired two-tailed t test). Note that PE1, PE2, PE3 and PE7 can shorten the CLS of WT yeast under non-CR conditions if added at high concentrations (n = 6; $*p < 0.05$; the p values for comparing the means of two groups were calculated with the help of the GraphPad Prism statistics software using an unpaired two-tailed t test).

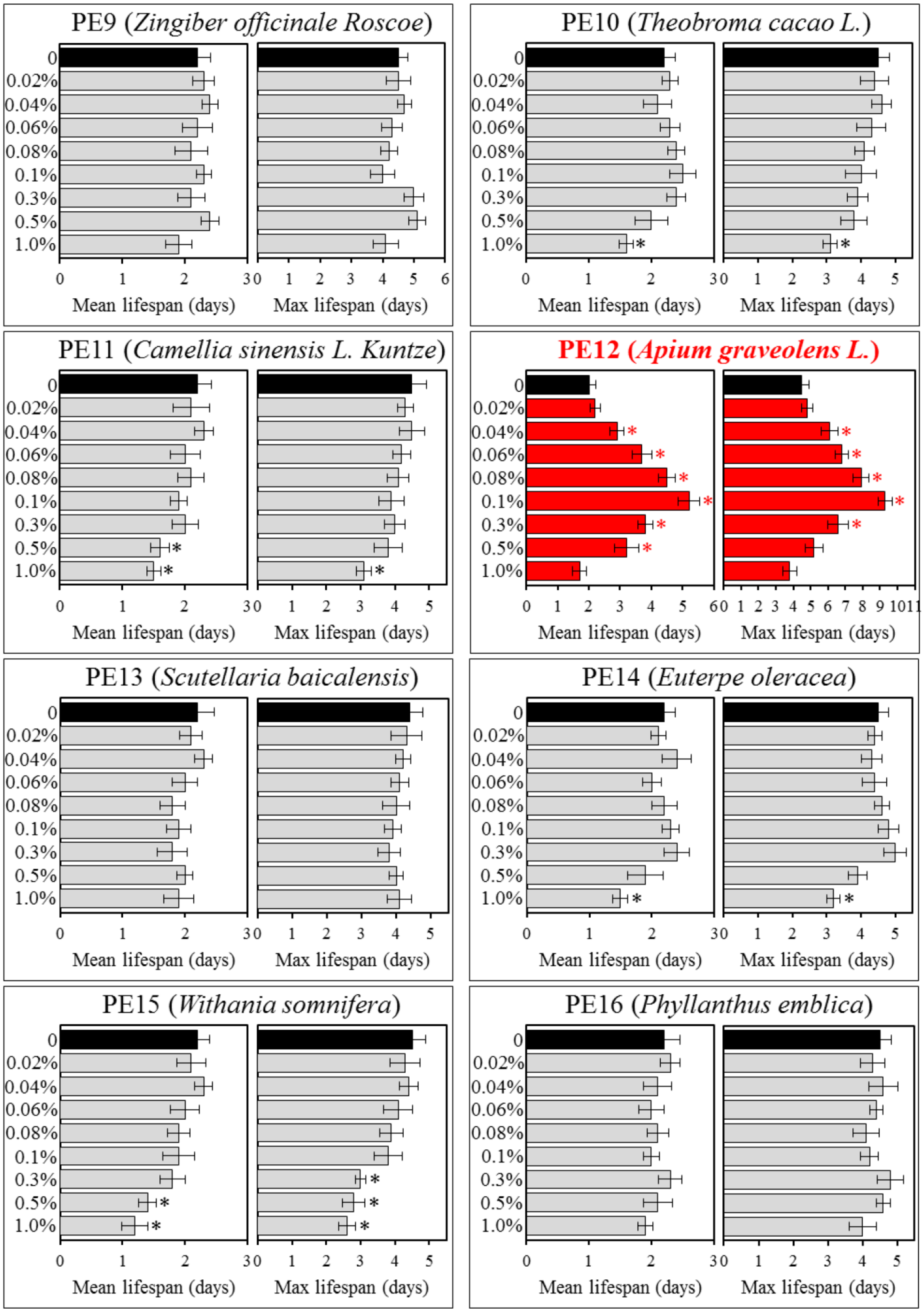

Supplementary Figure S2: PE12, but not PE9, PE10, PE11, PE13, PE14, PE15 or PE16, extends the CLS of WT yeast grown under non-CR conditions. WT cells were grown in the synthetic minimal YNB medium initially containing 2% glucose (non-CR conditions), in the presence of a PE or in its absence. The mean and maximum lifespans of chronologically aging WT strain cultured under non-CR conditions without a PE or with a PE added at various concentrations are shown; data are presented as means ± SEM (n = 6–29; *$p < 0.05$; the p values for comparing the means of two groups were calculated with the help of the GraphPad Prism statistics software using an unpaired two-tailed t test). Note that PE10, PE11, PE14 and PE15 can shorten the CLS of WT yeast under non-CR conditions if added at high concentrations (n = 6; *$p < 0.05$; the p values for comparing the means of two groups were calculated with the help of the GraphPad Prism statistics software using an unpaired two-tailed t test).

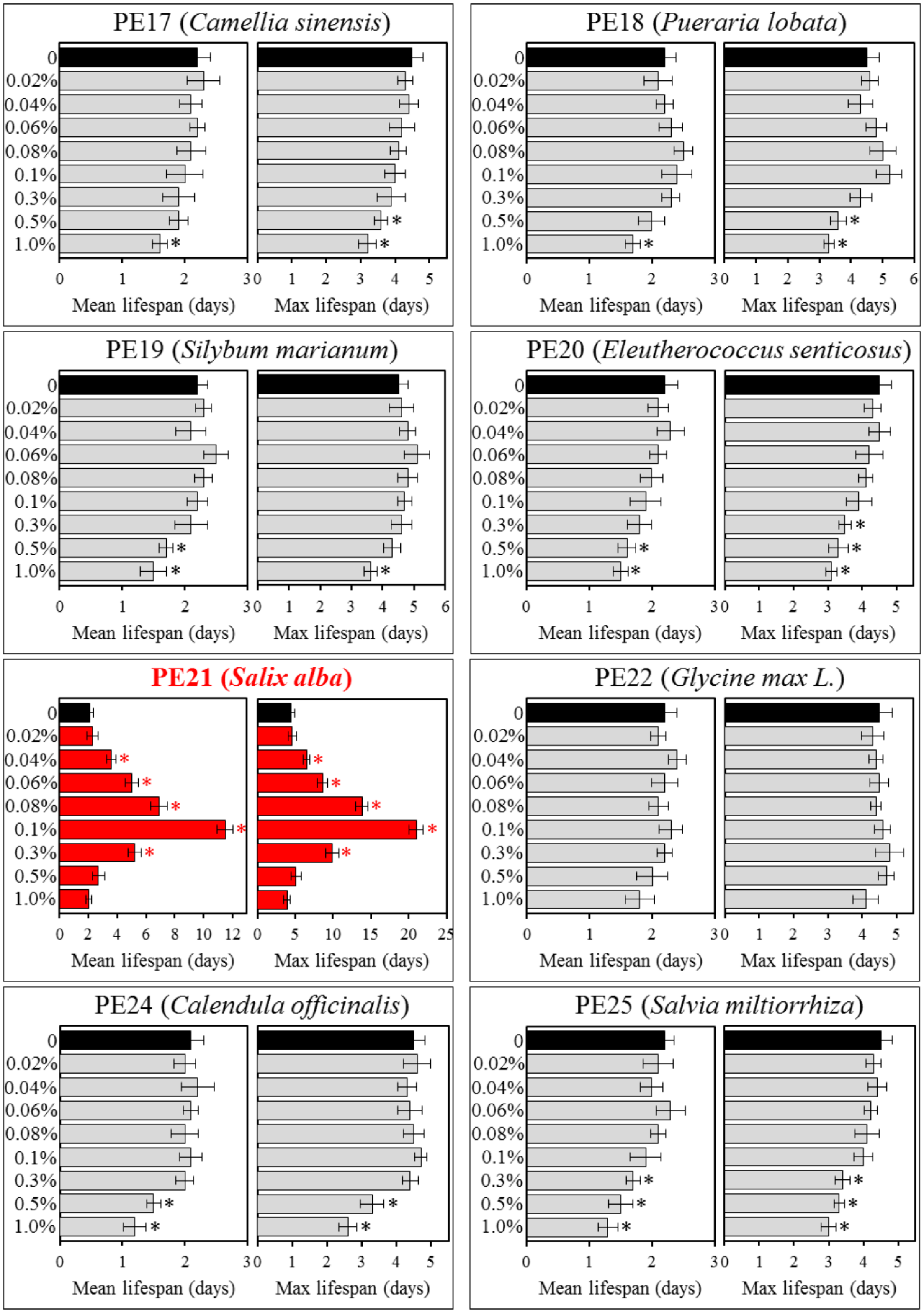

Supplementary Figure S3: PE21, but not PE17, PE18, PE19, PE20, PE22, PE24 or PE25, extends the CLS of WT yeast grown under non-CR conditions. WT cells were grown in the synthetic minimal YNB medium initially containing 2% glucose (non-CR conditions), in the presence of a PE or in its absence. The mean and maximum lifespans of chronologically aging WT strain cultured under non-CR conditions without a PE or with a PE added at various concentrations are shown; data are presented as means ± SEM (n = 6–35; *p < 0.05; the p values for comparing the means of two groups were calculated with the help of the GraphPad Prism statistics software using an unpaired two-tailed t test). Note that PE17, PE18, PE19, PE20, PE24 and PE25 can shorten the CLS of WT yeast under non-CR conditions if added at high concentrations (n = 6; *p < 0.05; the p values for comparing the means of two groups were calculated with the help of the GraphPad Prism statistics software using an unpaired two-tailed t test).

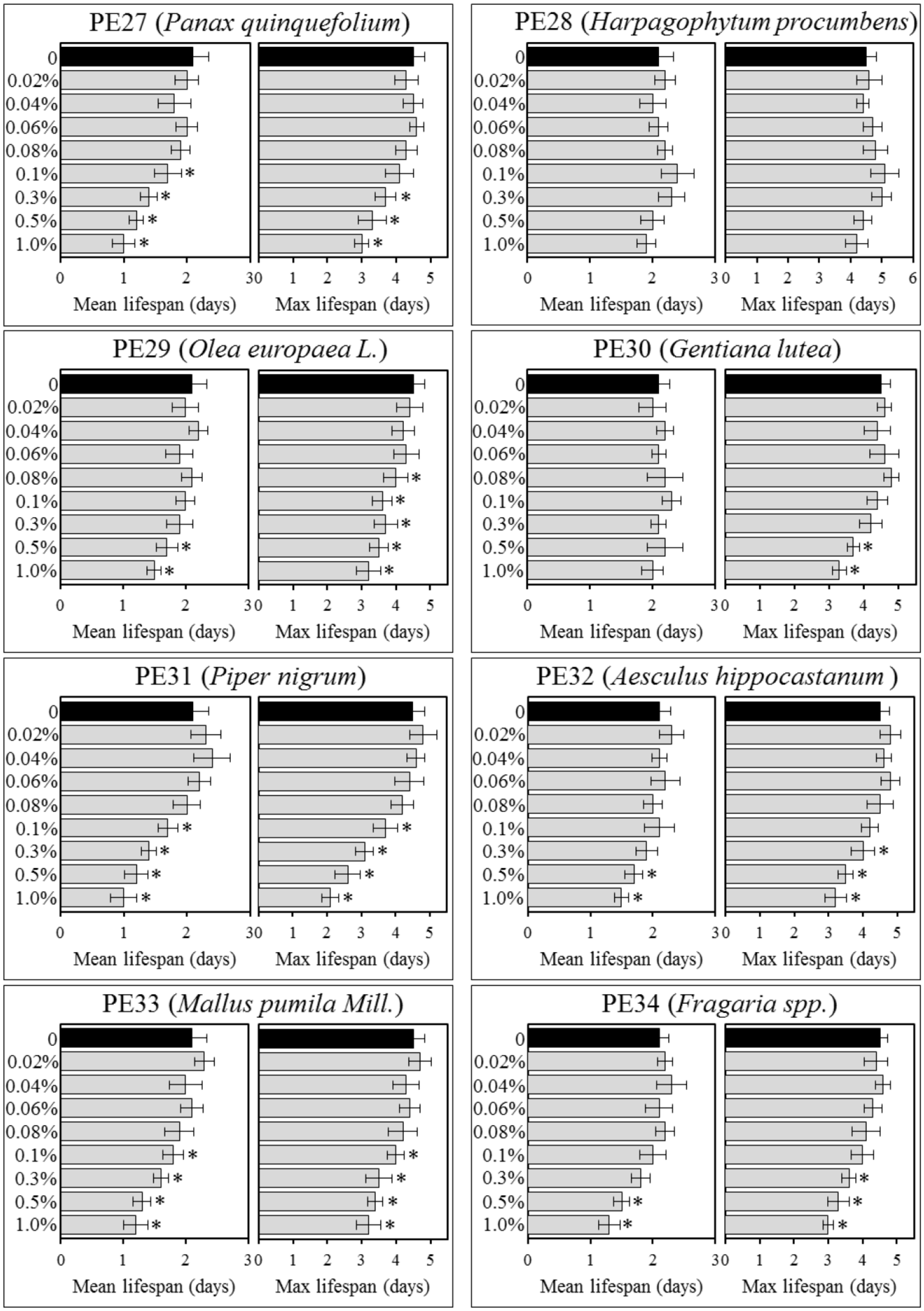

Supplementary Figure S4: PE27, PE28, PE29, PE30, PE31, PE32, PE33 and PE34 do not extend the CLS of WT yeast grown under non-CR conditions. WT cells were grown in the synthetic minimal YNB medium initially containing 2% glucose (non-CR conditions), in the presence of a PE or in its absence. The mean and maximum lifespans of chronologically aging WT strain cultured under non-CR conditions without a PE or with a PE added at various concentrations are shown; data are presented as means ± SEM (n = 5–6; *$p < 0.05$; the p values for comparing the means of two groups were calculated with the help of the GraphPad Prism statistics software using an unpaired two-tailed t test). Note that PE 27, PE29, PE30, PE31, PE32, PE33 and PE34 can shorten the CLS of WT yeast under non-CR conditions if added at high concentrations (n = 6; *$p < 0.05$; the p values for comparing the means of two groups were calculated with the help of the GraphPad Prism statistics software using an unpaired two-tailed t test).

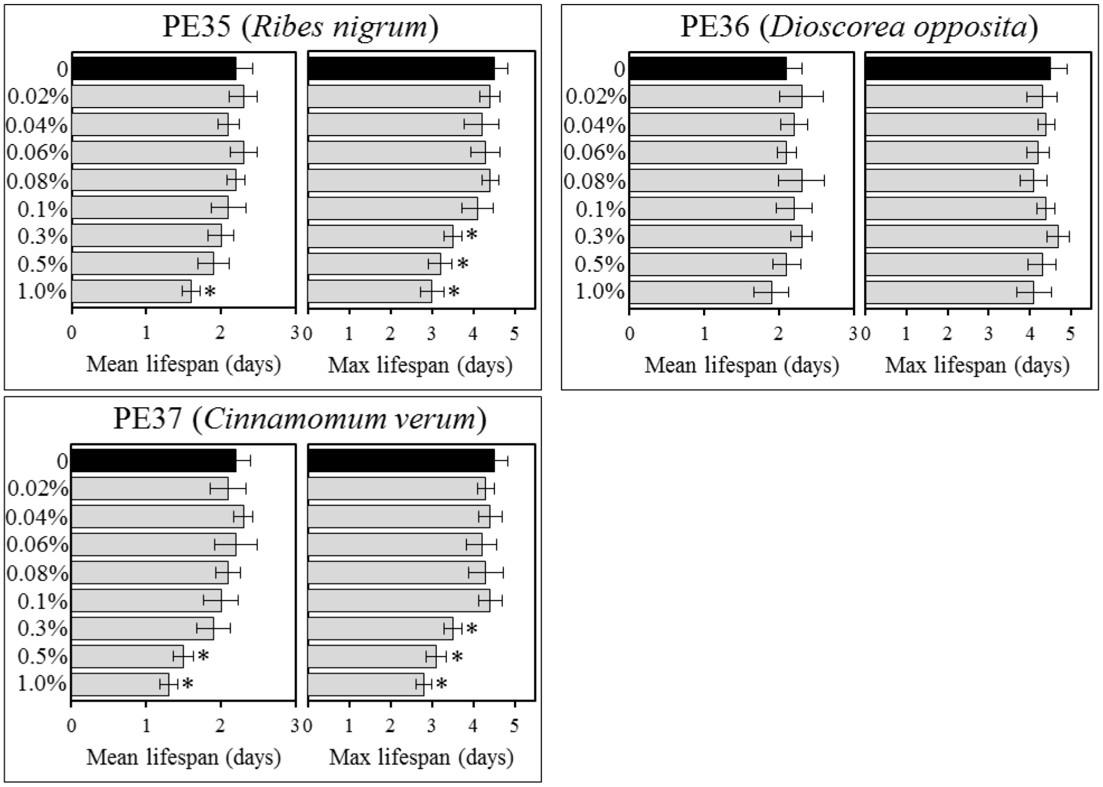

Supplementary Figure S5: PE35, PE36 and PE37 do not extend the CLS of WT yeast grown under non-CR conditions. WT cells were grown in the synthetic minimal YNB medium initially containing 2% glucose (non-CR conditions), in the presence of a PE or in its absence. The mean and maximum lifespans of chronologically aging WT strain cultured under non-CR conditions without a PE or with a PE added at various concentrations are shown; data are presented as means ± SEM (n = 5–6; *p < 0.05; the p values for comparing the means of two groups were calculated with the help of the GraphPad Prism statistics software using an unpaired two-tailed t test). Note that PE 35 and PE37 can shorten the CLS of WT yeast under non-CR conditions if added at high concentrations (n = 6; *p < 0.05; the p values for comparing the means of two groups were calculated with the help of the GraphPad Prism statistics software using an unpaired two-tailed t test).

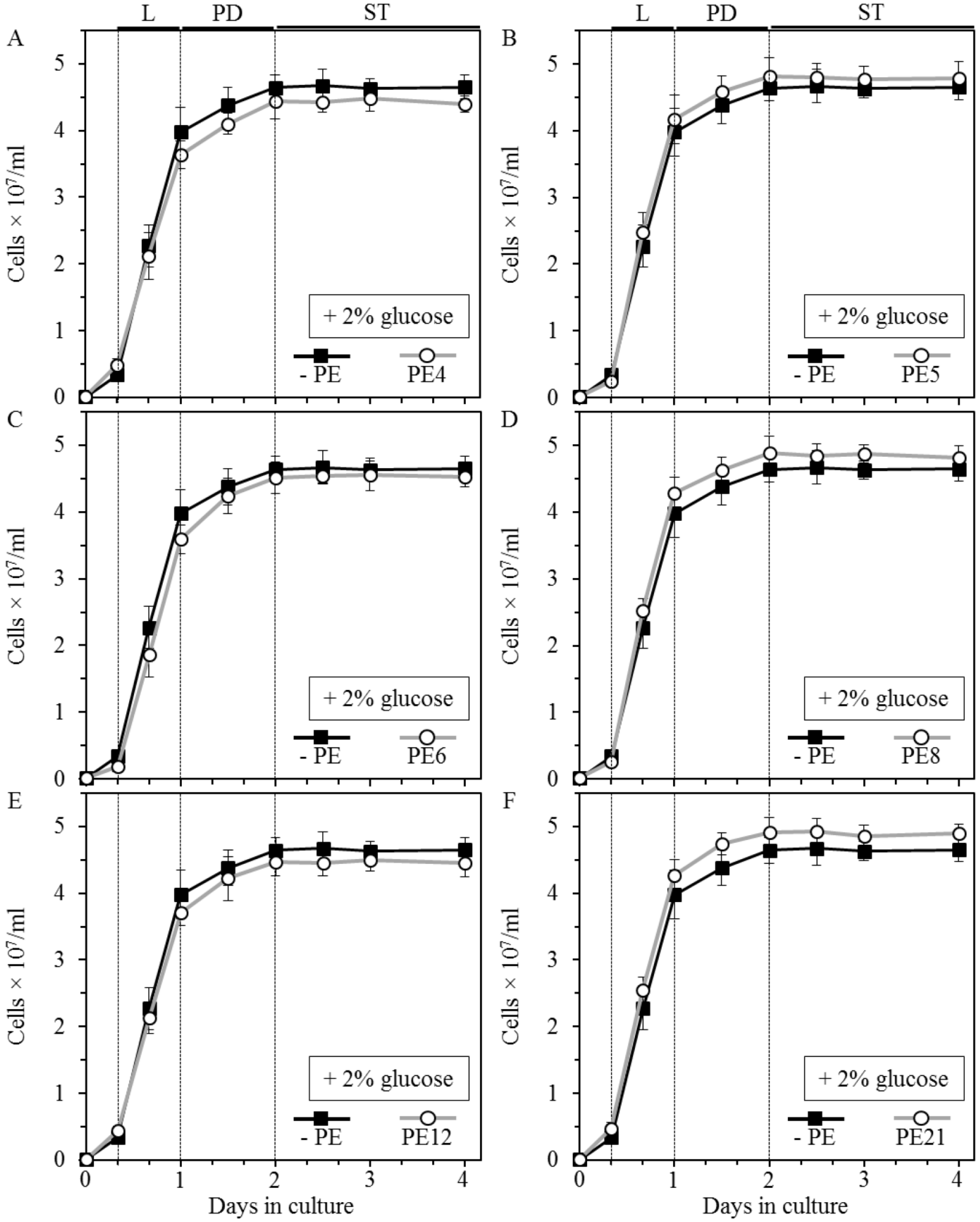

Supplementary Figure S6: PE4, PE5, PE6, PE8, PE12 and PE21 do not cause significant effects on growth of WT yeast under non-CR conditions. WT cells were grown in the synthetic minimal YNB medium initially containing 2% glucose (non-CR conditions), in the absence of a PE or in the presence of 0.5% PE4 (**A**), 0.5% PE5 (**B**), 1% PE6 (**C**), 0.3% PE8 (**D**), 0.1% PE12 (**E**) or 0.1% PE21 (**F**). Kinetics of cell growth is shown (*n* = 8–14). Abbreviations: Logarithmic (L), post-diauxic (PD) or stationary (ST) growth phase.

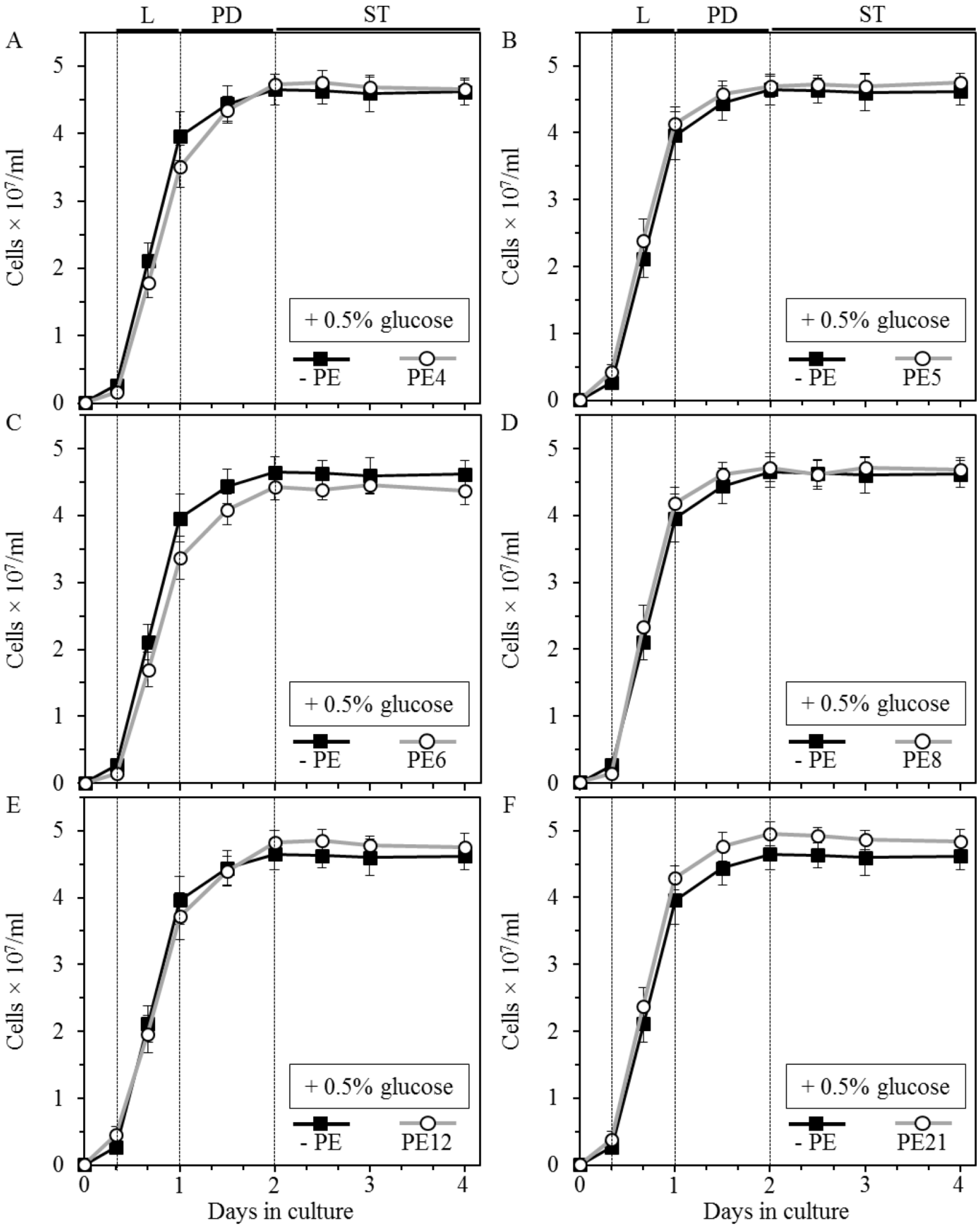

Supplementary Figure S7: PE4, PE5, PE6, PE8, PE12 and PE21 do not cause significant effects on growth of WT yeast under CR conditions. WT cells were grown in the synthetic minimal YNB medium initially containing 0.5% glucose (CR conditions), in the absence of a PE or in the presence of 0.5% PE4 (**A**), 0.5% PE5 (**B**), 1% PE6 (**C**), 0.3% PE8 (**D**), 0.1% PE12 (**E**) or 0.1% PE21 (**F**). Kinetics of cell growth is shown (*n* = 6–9). Abbreviations: Logarithmic (L), post-diauxic (PD) or stationary (ST) growth phase.

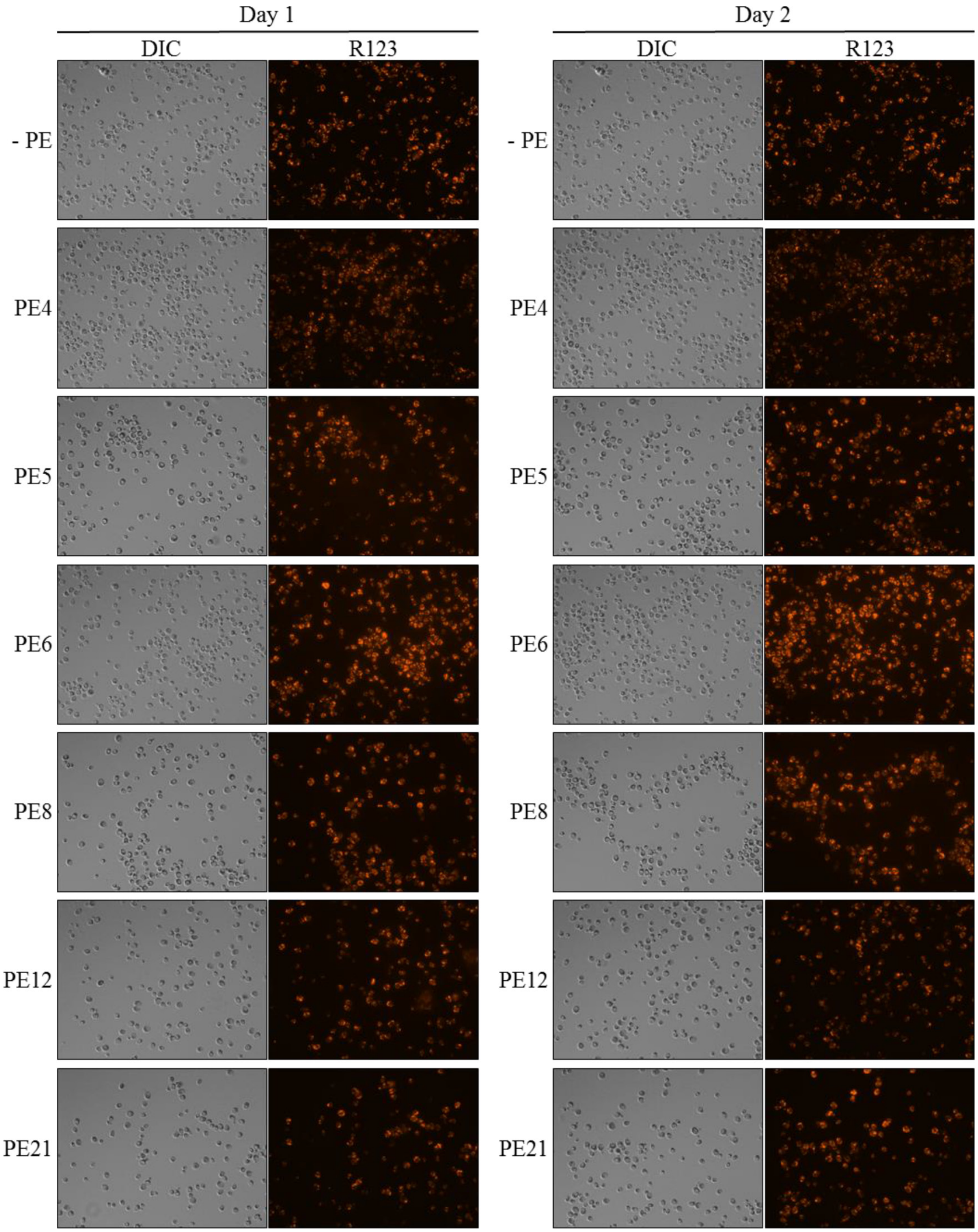

Supplementary Figure S8: PE4, PE5, PE6, PE8, PE12 and PE21 significantly delay an age-dependent decline in the number of WT cells that exhibit high mitochondrial membrane potential under non-CR conditions. WT cells were grown in the synthetic minimal YNB medium initially containing 2% glucose, in the presence of a PE or in its absence. Yeast cells were recovered at days 1 and 2 of culturing, stained with Rhodamine 123 (R123) for visualizing cells displaying high mitochondrial membrane potential, and subjected to live-cell fluorescence microscopy and differential interference contrast (DIC) microscopy as described in Materials and methods.

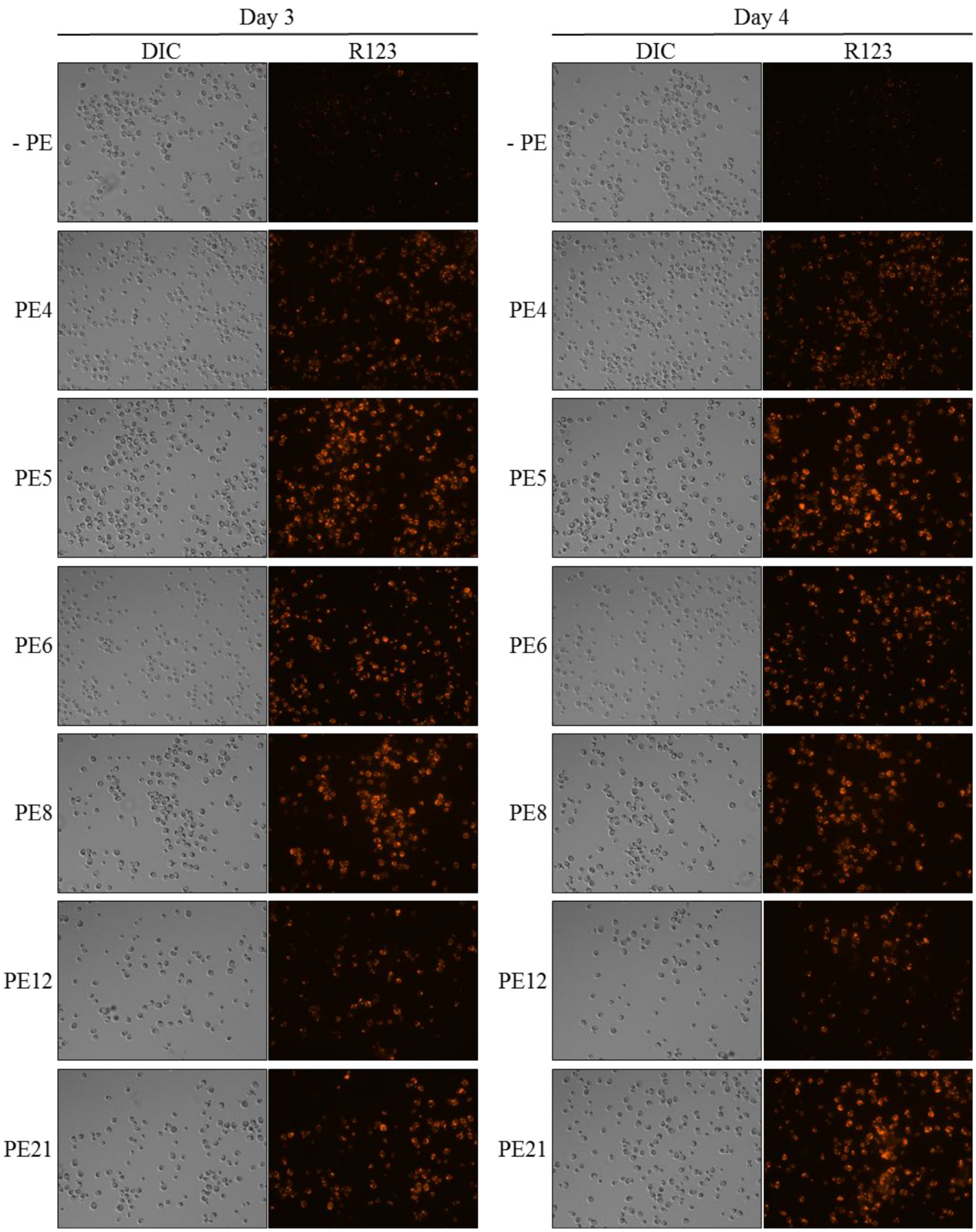

Supplementary Figure S9: PE4, PE5, PE6, PE8, PE12 and PE21 significantly delay an age-dependent decline in the number of WT cells that exhibit high mitochondrial membrane potential under non-CR conditions. WT cells were grown in the synthetic minimal YNB medium initially containing 2% glucose, in the presence of a PE or in its absence. Yeast cells were recovered at days 3 and 4 of culturing, stained with R123 for visualizing cells displaying high mitochondrial membrane potential, and subjected to live-cell fluorescence microscopy and DIC microscopy as described in Materials and methods.

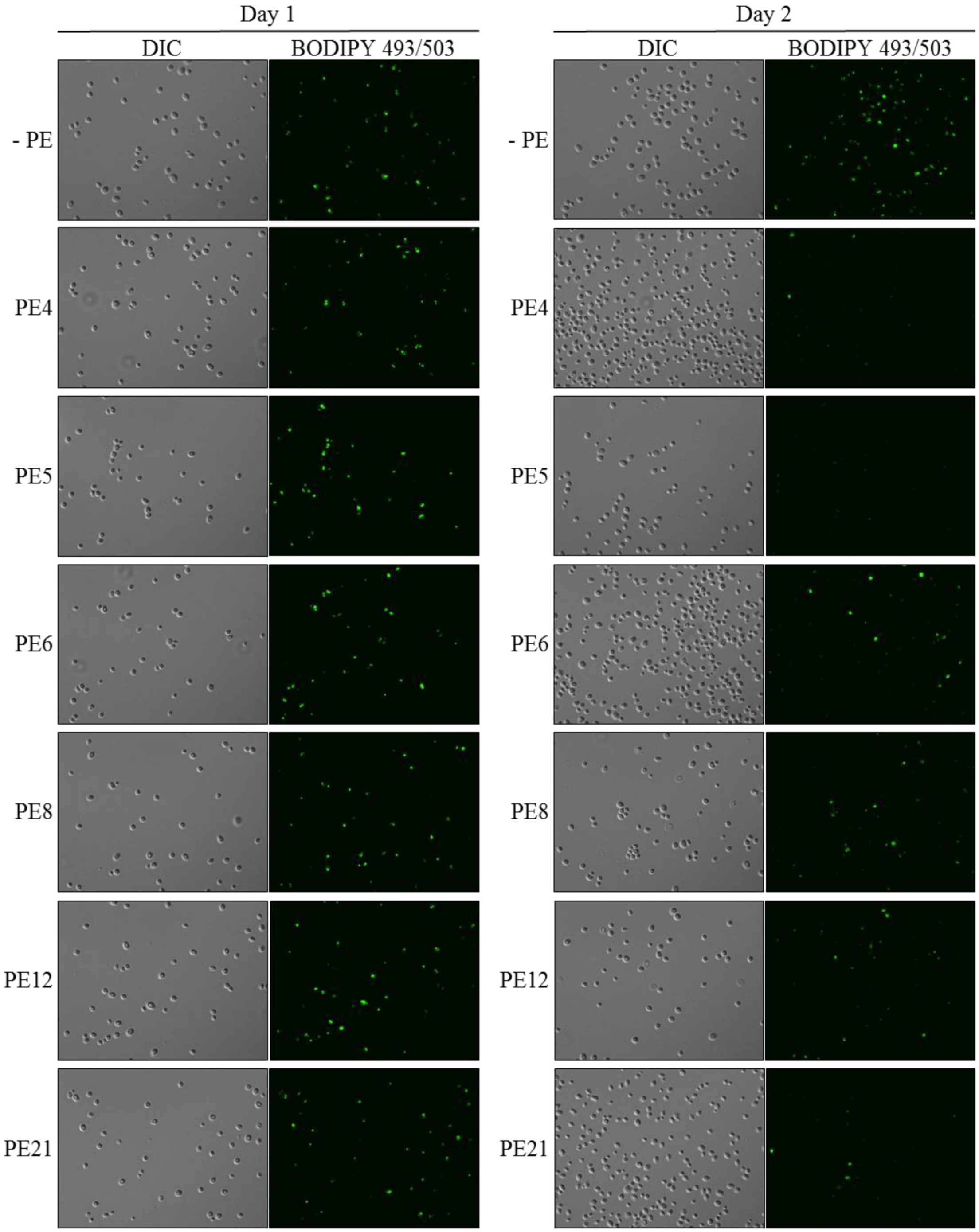

Supplementary Figure S10: PE4, PE5, PE6, PE8, PE12 and PE21 significantly accelerate an age-dependent decline in the number of WT cells that exhibit LDs under non-CR conditions. WT cells were grown in the synthetic minimal YNB medium initially containing 2% glucose, in the presence of a PE or in its absence. Yeast cells were recovered at days 1 and 2 of culturing, stained with BODIPY 493/503 for visualizing cells displaying neutral lipids deposited in LDs, and subjected to live-cell fluorescence microscopy and differential interference contrast (DIC) microscopy as described in Materials and methods.

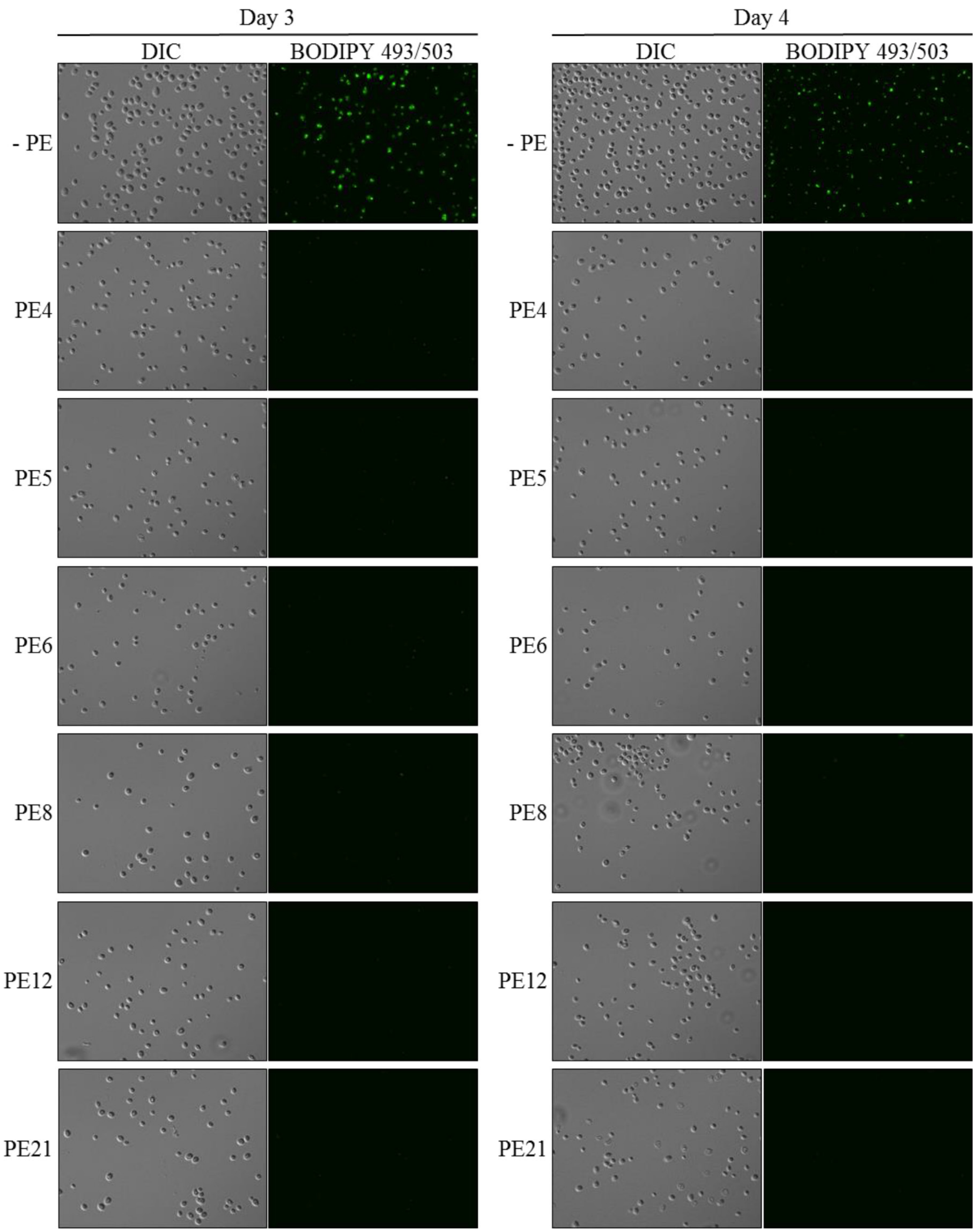

Supplementary Figure S11: PE4, PE5, PE6, PE8, PE12 and PE21 significantly accelerate an age-dependent decline in the number of WT cells that exhibit LDs under non-CR conditions. WT cells were grown in the synthetic minimal YNB medium initially containing 2% glucose, in the presence of a PE or in its absence. Yeast cells were recovered at days 3 and 4 of culturing, stained with BODIPY 493/503 for visualizing cells displaying neutral lipids deposited in LDs, and subjected to live-cell fluorescence microscopy and differential interference contrast (DIC) microscopy as described in Materials and methods.

Supplementary Table S1: Percent increase of lifespan by geroprotective PEs discovered in this study and by longevity-extending chemical compounds that have been previously identified

PE or chemical compound	% increase of lifespan* [reference]	Organism exhibiting lifespan increase
0.5% PE4	195% (mean CLS) [this study]<br>100% (max** CLS) [this study]	*S. cerevisiae*
0.5% PE5	185% (mean CLS) [this study]<br>87% (max CLS) [this study]	*S. cerevisiae*
1.0 % PE6	180% (mean CLS) [this study]<br>80% (max CLS) [this study]	*S. cerevisiae*
0.3% PE8	145% (mean CLS) [this study]<br>104% (max) [this study]	*S. cerevisiae*
0.1% PE12	160% (mean CLS) [this study]<br>107% (max CLS) [this study]	*S. cerevisiae*
0.1% PE21	475% (mean CLS) [this study]<br>369% (max CLS) [this study]	*S. cerevisiae*
Acteoside	24–25% (female); 16–18% (male) (mean OLS) [1]<br>9–13% (female); 9–15% (male) (max OLS) [1]	The fruit fly *Drosophila melanogaster*
Butein	55% (mean RLS) [2]	*S. cerevisiae*
Caffeic acid	11% (mean OLS) [3]	The nematode *Caenorhabditis elegans*
Caffeine	8% (median CLS) [4]	*S. cerevisiae*
	46% (mean CLS) [5]<br>17% (max CLS) [5]	The yeast *Schizosaccharomyces pombe*
	52% (mean OLS) [6]; 37% (mean OLS) [7]<br>29% (median OLS) [6]	*C. elegans*
Catechin	15% (mean OLS) [8]<br>14% (median OLS) [8]	*C. elegans*
Celastrol	13% (mean OLS) [9]	Mouse model of amyotrophic lateral sclerosis
Curcumin, tetrahydrocurcumin	13%–39% (mean OLS) [10]	*C. elegans*
	26% (females; mean OLS); 16% (males; mean OLS) [11, 12] 75% (median OLS) [13]	*D. melanogaster*, including 5 different models of the Alzheimer's disease
Crocin	37%–58% (max OLS) [14]	Dalton's lymphoma ascites bearing mice
Cryptotanshinone	34% (mean CLS) [15]	*S. cerevisiae*
Cyanidin	2%–6% (max RLS; untreated cells); 14–21% (max RLS; prematurely aging cells pre-treated with H_2O_2) [16]	WI-38 human diploid fibroblasts
Diallyl trisulfide	13% (mean OLS) [17]	*C. elegans*

Ellagic acid	9% (mean OLS); 11%–13% (median OLS) [18]	*C. elegans*
Epigallocatechin gallate	10%–14% (mean OLS) [19]	*C. elegans*
Epicatechin	42% (mean OLS) [20]<br>3% (max OLS) [20]	*D. melanogaster*
	8% (mean OLS) [20]	Obese diabetic mice
Ferulsinaic acid	18% (mean CLS) [21]<br>42% (max OLS) [21]	*C. elegans*
Fisetin	31% (mean RLS) [2]	*S. cerevisiae*
	6% (mean OLS) [22]<br>6% (median OLS) [22]	*C. elegans*
Fucoxanthin	14% (mean OLS) [23]<br>24% (max OLS) [23]	*C. elegans*
	33%– 49% (females; median OLS); 33% (males; median OLS) [23]<br>22%–27% (females; max OLS); 12%–17% (males; max OLS) [23]	*D. melanogaster*
Gallic acid	12% (mean OLS) [18]<br>14% (median OLS) [18]	*C. elegans*
HDTIC-1, HDTIC-2	14%–38% (max RLS) [24]	Human fetal lung diploid fibroblasts
Icariin, icariside II	31% (mean OLS) [25]	*C. elegans*
Kaempferol	10% (mean OLS) [22]<br>6% (median OLS) [22]	*C. elegans*
Lipoic acid	21% (median OLS) [26]	*C. elegans*
	12% (females; average OLS); 15% (females; median OLS) [27] 4% (males; average OLS);<br>4% (males; median OLS) [27]	*D. melanogaster*
Lithocholic acid	146% (mean CLS) [28, 29]<br>100% (max CLS) [28, 29]	*S. cerevisiae*
Metformin	40% (median OLS) [30]	*C. elegans*
	38% (mean OLS) [31]<br>10% (max OLS) [31]	Mice
Methionine sulfoximine	78% (mean CLS) [32]<br>63% (max CLS) [32]	*S. cerevisiae*
Mianserin	25% (mean OLS) [33]	*C. elegans*
Myricetin	15% (mean OLS) [34]<br>17% (median OLS) [34]<br>24% (max OLS) [34]	*C. elegans*

Nordihydroguaiaretic acid	10% (median OLS) [35]<br>32% (max OLS) [35]	Mouse model of the Alzheimer’s disease
	12% (median OLS) [36]	Male mice
	12% (mean OLS) [37]	*D. melanogaster*
	42%–64% (mean OLS) [38]	Mosquitoes
Oleuropein	15% (max RLS) [39]	Human embryonic fibroblasts
Phloridzin	35% (mean RLS) [40]<br>41% (max RLS) [40]	*S. cerevisiae*
Propyl gallate	41% (median OLS) [26]	*C. elegans*
Quercetin	60% (mean CLS) [41]	*S. cerevisiae*
	15% (mean OLS) [42]<br>18% (mean OLS) [43]<br>14% (median OLS) [44]	*C. elegans*
	5% (max RLS) [45]	Human embryonic fibroblasts
Rapamycin	16% (mean RLS) [46]<br>36% (mean CLS) [47]	*S. cerevisiae*
	17% (mean OLS) [48]<br>23% (max OLS) [48]	*D. melanogaster*
	13% (females; mean OLS); 9% (males; mean OLS) [49]<br>14% (females; max OLS); 9% (males; max OLS) [49]	Mice
Reserpine	64% (mean OLS); 50% (max OLS) [50]	*C. elegans*
	52% (mean OLS) [51]	*C. elegans* model of the Alzheimer’s disease
Resveratrol	61% (mean RLS) [2]	*S. cerevisiae*
	10% (mean OLS) [52]	*C. elegans*
	20% (females; mean OLS); 16% (males; mean OLS) [52]	*D. melanogaster*
	56% (median OLS) [53]<br>59% (max OLS) [53]	The short-lived fish *Nothobranchius furzeri*
	38% (mean OLS) [54]	The honey bee *Apis mellifera*
	4% (max OLS) [55]	Mice on a high-calorie diet
Rosmarinic acid	10% (mean OLS) [3]	*C. elegans*

SkQ1	38% (mean CLS) [56]<br>16% (max CLS) [56]	The fungus *Podospora anserina*
	69% (mean CLS) [56]<br>64% (max CLS) [56]	The crustacean *Ceriodaphnia affinis*
	13% (females; mean OLS) [56]<br>7% (females; max OLS) [56]	*D. melanogaster*
	43% (mean OLS) [56]<br>58% (max OLS) [56]	p53-Deficient mice
	52% (mean OLS) [56]<br>34% (max OLS) [56]	Tumor-bearing immunodeficient mice
Sodium nitroprusside	60% (max RLS) [57]	Human peripheral blood mononuclear cells
Spermidine	200% (mean CLS) [58]<br>183% (mean RLS) [58]<br>17% (max RLS) [58]	*S. cerevisiae*
	15% (mean OLS) [58]<br>14% (max) OLS) [58]	*C. elegans*
	30% (mean OLS) [58]<br>8% (max OLS) [58]	*D. melanogaster*
	178% (max RLS) [58]	Human peripheral blood mononuclear cells
Tannic acid	18%–25% (mean OLS) [6, 18, 59]; 18% (median OLS) [18, 59]<br>59% (max OLS) [6]	*C. elegans*
Taxifolin	26% (median OLS) [26]	*C. elegans*
Trolox	15% (median OLS) [26]	*C. elegans*
Tyrosol	21% (mean OLS); 21% (median OLS) [60]<br>11% (maximum OLS) [60]	*C. elegans*
Valproic acid	35% (mean OLS) [61]<br>42% (max OLS) [61]	*C. elegans*

*Chronological or replicative lifespan (CLS or RLS, respectively) of cell cultures or organismal lifespan (OLS).

**Abbreviation: max, maximum.

REFERENCES

1. Pan W, Jiang S, Luo P, Wu J, Gao P. Isolation, purification and structure identification of antioxidant compound from the roots of Incarvillea younghusbandii Sprague and its life span prolonging effect in Drosophila melanogaster. Nat Prod Res. 2008; 22:719–725.
2. Howitz KT, Bitterman KJ, Cohen HY, Lamming DW, Lavu S, Wood JG, Zipkin RE, Chung P, Kisielewski A, Zhang LL, Scherer B, Sinclair DA. Small molecule activators of sirtuins extend Saccharomyces cerevisiae lifespan. Nature. 2003; 425:191–196.
3. Pietsch K, Saul N, Chakrabarti S, Stürzenbaum SR, Menzel R and Steinberg CE. Hormetins, antioxidants and prooxidants: defining quercetin-, caffeic acid- and rosmarinic acid-mediated life extension in C. elegans. Biogerontology. 2011; 12:329–347.
4. Wanke V, Cameroni E, Uotila A, Piccolis M, Urban J, Loewith R, De Virgilio C. Caffeine extends yeast lifespan by targeting TORC1. Mol Microbiol. 2008; 69:277–285.
5. Rallis C, Codlin S, Bähler J. TORC1 signaling inhibition by rapamycin and caffeine affect lifespan, global gene expression, and cell proliferation of fission yeast. Aging Cell. 2013; 12:563–573.
6. Lublin A, Isoda F, Patel H, Yen K, Nguyen L, Hajje D, Schwartz M, Mobbs C. FDA-approved drugs that protect mammalian neurons from glucose toxicity slow aging dependent on cbp and protect against proteotoxicity. PLoS One. 2011; 6:e27762.
7. Sutphin GL, Bishop E, Yanos ME, Moller RM, Kaeberlein M. Caffeine extends life span, improves healthspan, and delays age-associated pathology in Caenorhabditis elegans. Longev Healthspan. 2012; 1:9.
8. Saul N, Pietsch K, Menzel R, Stürzenbaum SR, Steinberg CE. Catechin induced longevity in C. elegans: from key regulator genes to disposable soma. Mech Ageing Dev. 2009; 130:477–486.
9. Kiaei M, Kipiani K, Petri S, Chen J, Calingasan NY, Beal MF. Celastrol blocks neuronal cell death and extends life in transgenic mouse model of amyotrophic lateral sclerosis. Neurodegener Dis. 2005; 2:246–254.
10. Liao VH, Yu CW, Chu YJ, Li WH, Hsieh YC, Wang TT. Curcumin-mediated lifespan extension in Caenorhabditis elegans. Mech Ageing Dev. 2011; 132:480–487.
11. Lee KS, Lee BS, Semnani S, Avanesian A, Um CY, Jeon HJ, Seong KM, Yu K, Min KJ, Jafari M. Curcumin extends life span, improves health span, and modulates the expression of age-associated aging genes in Drosophila melanogaster. Rejuvenation Res. 2010; 13:561–570.
12. Shen LR, Xiao F, Yuan P, Chen Y, Gao QK, Parnell LD, Meydani M, Ordovas JM, Li D, Lai CQ. Curcumin-supplemented diets increase superoxide dismutase activity and mean lifespan in Drosophila. Age (Dordr). 2013; 35:1133–1142.
13. Caesar I, Jonson M, Nilsson KP, Thor S, Hammarström P. Curcumin promotes A-beta fibrillation and reduces neurotoxicity in transgenic Drosophila. PLoS One. 2012; 7:e31424.
14. Bakshi HA, Sam S, Feroz A, Ravesh Z, Shah GA, Sharma M. Crocin from Kashmiri saffron (Crocus sativus) induces in vitro and in vivo xenograft growth inhibition of Dalton's lymphoma (DLA) in mice. Asian Pac J Cancer Prev. 2009; 10:887–890.
15. Wu Z, Song L, Liu SQ, Huang D. Tanshinones extend chronological lifespan in budding yeast Saccharomyces cerevisiae. Appl Microbiol Biotechnol. 2014; 98:8617–8628.
16. Choi MJ, Kim BK, Park KY, Yokozawa T, Song YO, Cho EJ. Anti-aging effects of cyanidin under a stress-induced premature senescence cellular system. Biol Pharm Bull. 2010; 33:421–426.
17. Powolny AA, Singh SV, Melov S, Hubbard A, Fisher AL. The garlic constituent diallyl trisulfide increases the lifespan of C. elegans via skn-1 activation. Exp Gerontol. 2011; 46:441–452.
18. Saul N, Pietsch K, Stürzenbaum SR, Menzel R, Steinberg CE. Diversity of polyphenol action in Caenorhabditis elegans: between toxicity and longevity. J Nat Prod. 2011; 74:1713–1720.
19. Abbas S, Wink M. Epigallocatechin gallate from green tea (Camellia sinensis) increases lifespan and stress resistance in Caenorhabditis elegans. Planta Med. 2009; 75:216–221.
20. Si H, Fu Z, Babu PV, Zhen W, Leroith T, Meaney MP, Voelker KA, Jia Z, Grange RW, Liu D. Dietary epicatechin promotes survival of obese diabetic mice and Drosophila melanogaster. J Nutr. 2011; 141:1095–1100.
21. Sayed AA. Ferulsinaic acid attenuation of advanced glycation end products extends the lifespan of Caenorhabditis elegans. J Pharm Pharmacol. 2011; 63:423–428.
22. Kampkötter A, Gombitang Nkwonkam C, Zurawski RF, Timpel C, Chovolou Y, Wätjen W, Kahl R. Effects of the flavonoids kaempferol and fisetin on thermotolerance, oxidative stress and FoxO transcription factor DAF-16 in the model organism Caenorhabditis elegans. Arch Toxicol. 2007; 81:849–858.
23. Lashmanova E, Proshkina E, Zhikrivetskaya S, Shevchenko O, Marusich E, Leonov S, Melerzanov A, Zhavoronkov A, Moskalev A. Fucoxanthin increases lifespan of Drosophila melanogaster and Caenorhabditis elegans. Pharmacol Res. 2015; 100:228–241.
24. Wang P, Zhang Z, Ma X, Huang Y, Liu X, Tu P, Tong T. HDTIC-1 and HDTIC-2, two compounds extracted from Astragali Radix, delay replicative senescence of human diploid fibroblasts. Mech Ageing Dev. 2003; 124:1025–1034.
25. Cai WJ, Huang JH, Zhang SQ, Wu B, Kapahi P, Zhang XM, Shen ZY. Icariin and its derivative icariside II extend healthspan via insulin/IGF-1 pathway in C. elegans. PLoS One. 2011; 6:e28835.

26. Benedetti MG, Foster AL, Vantipalli MC, White MP, Sampayo JN, Gill MS, Olsen A, Lithgow GJ. Compounds that confer thermal stress resistance and extended lifespan. Exp Gerontol. 2008; 43:882–891.
27. Bauer JH, Goupil S, Garber GB, Helfand SL. An accelerated assay for the identification of lifespan-extending interventions in Drosophila melanogaster. Proc Natl Acad Sci USA. 2004; 101:12980–12985.
28. Goldberg AA, Richard VR, Kyryakov P, Bourque SD, Beach A, Burstein MT, Glebov A, Koupaki O, Boukh-Viner T, Gregg C, Juneau M, English AM, Thomas DY, et al. Chemical genetic screen identifies lithocholic acid as an anti-aging compound that extends yeast chronological life span in a TOR-independent manner, by modulating housekeeping longevity assurance processes. Aging (Albany NY). 2010; 2:393–414.
29. Beach A, Richard VR, Leonov A, Burstein MT, Bourque SD, Koupaki O, Juneau M, Feldman R, Iouk T, Titorenko VI. Mitochondrial membrane lipidome defines yeast longevity. Aging (Albany NY). 2013; 5:551–574.
30. Onken B, Driscoll M. Metformin induces a dietary restriction-like state and the oxidative stress response to extend C. elegans healthspan via AMPK, LKB1, and SKN-1. PLoS One. 2010; 5:e8758.
31. Anisimov VN, Berstein LM, Egormin PA, Piskunova TS, Popovich IG, Zabezhinski MA, Tyndyk ML, Yurova MV, Kovalenko IG, Poroshina TE, Semenchenko AV. Metformin slows down aging and extends life span of female SHR mice. Cell Cycle. 2008; 7:2769–2773.
32. Powers RW 3rd, Kaeberlein M, Caldwell SD, Kennedy BK, Fields S. Extension of chronological life span in yeast by decreased TOR pathway signaling. Genes Dev. 2006; 20:174–184.
33. Petrascheck M, Ye X, Buck LB. An antidepressant that extends lifespan in adult Caenorhabditis elegans. Nature. 2007; 450:553–556.
34. Grünz G, Haas K, Soukup S, Klingenspor M, Kulling SE, Daniel H, Spanier B. Structural features and bioavailability of four flavonoids and their implications for lifespan-extending and antioxidant actions in C. elegans. Mech Ageing Dev. 2012; 133:1–10.
35. West M, Mhatre M, Ceballos A, Floyd RA, Grammas P, Gabbita SP, Hamdheydari L, Mai T, Mou S, Pye QN, Stewart C, West S, Williamson KS, et al. The arachidonic acid 5-lipoxygenase inhibitor nordihydroguaiaretic acid inhibits tumor necrosis factor alpha activation of microglia and extends survival of G93A-SOD1 transgenic mice. J Neurochem. 2004; 91:133–143.
36. Strong R, Miller RA, Astle CM, Floyd RA, Flurkey K, Hensley KL, Javors MA, Leeuwenburgh C, Nelson JF, Ongini E, Nadon NL, Warner HR, Harrison DE. Nordihydroguaiaretic acid and aspirin increase lifespan of genetically heterogeneous male mice. Aging Cell. 2008; 7:641–650.
37. Miquel J, Fleming J, Economos AC. Antioxidants, metabolic rate and aging in Drosophila. Arch Gerontol Geriatr. 1982; 1:159–165.
38. Richie JP Jr, Mills BJ, Lang CA. Dietary nordihydroguaiaretic acid increases the life span of the mosquito. Proc Soc Exp Biol Med. 1986; 183:81–85.
39. Katsiki M, Chondrogianni N, Chinou I, Rivett AJ, Gonos ES. The olive constituent oleuropein exhibits proteasome stimulatory properties in vitro and confers life span extension of human embryonic fibroblasts. Rejuvenation Res. 2007; 10:157–172.
40. Xiang L, Sun K, Lu J, Weng Y, Taoka A, Sakagami Y, Qi J. Anti-aging effects of phloridzin, an apple polyphenol, on yeast via the SOD and Sir2 genes. Biosci Biotechnol Biochem. 2011; 75:854–858.
41. Belinha I, Amorim MA, Rodrigues P, de Freitas V, Moradas-Ferreira P, Mateus N, Costa V. Quercetin increases oxidative stress resistance and longevity in Saccharomyces cerevisiae. J Agric Food Chem. 2007; 55:2446–2451.
42. Kampkötter A, Timpel C, Zurawski RF, Ruhl S, Chovolou Y, Proksch P, Wätjen W. Increase of stress resistance and lifespan of Caenorhabditis elegans by quercetin. Comp Biochem Physiol B Biochem Mol Biol. 2008; 149:314–323.
43. Pietsch K, Saul N, Menzel R, Stürzenbaum SR, Steinberg CE. Quercetin mediated lifespan extension in Caenorhabditis elegans is modulated by age-1, daf-2, sek-1 and unc-43. Biogerontology. 2009; 10:565–578.
44. Xue YL, Ahiko T, Miyakawa T, Amino H, Hu F, Furihata K, Kita K, Shirasawa T, Sawano Y, Tanokura M. Isolation and Caenorhabditis elegans lifespan assay of flavonoids from onion. J Agric Food Chem. 2011; 59:5927–5934.
45. Chondrogianni N, Kapeta S, Chinou I, Vassilatou K, Papassideri I, Gonos ES. Anti-ageing and rejuvenating effects of quercetin. Exp Gerontol. 2010; 45:763–771.
46. Medvedik O, Lamming DW, Kim KD, Sinclair DA. MSN2 and MSN4 link calorie restriction and TOR to sirtuin-mediated lifespan extension in Saccharomyces cerevisiae. PLoS Biol. 2007; 5:e261.
47. Bonawitz ND, Chatenay-Lapointe M, Pan Y, Shadel GS. Reduced TOR signaling extends chronological life span via increased respiration and upregulation of mitochondrial gene expression. Cell Metab. 2007; 5:265–277.
48. Bjedov I, Toivonen JM, Kerr F, Slack C, Jacobson J, Foley A, Partridge L. Mechanisms of life span extension by rapamycin in the fruit fly Drosophila melanogaster. Cell Metab. 2010; 11:35–46.
49. Harrison DE, Strong R, Sharp ZD, Nelson JF, Astle CM, Flurkey K, Nadon NL, Wilkinson JE, Frenkel K, Carter CS, Pahor M, Javors MA, Fernandez E, et al. Rapamycin fed late in life extends lifespan in genetically heterogeneous mice. Nature. 2009; 460:392–395.
50. Srivastava D, Arya U, SoundaraRajan T, Dwivedi H, Kumar S, Subramaniam JR. Reserpine can confer stress tolerance and lifespan extension in the nematode C. elegans. Biogerontology. 2008; 9:309–316.
51. rya U, Dwivedi H, Subramaniam JR. Reserpine ameliorates Abeta toxicity in the Alzheimer's disease model in Caenorhabditis elegans. Exp Gerontol. 2009; 44:462–466.

52. Wood JG, Rogina B, Lavu S, Howitz K, Helfand SL, Tatar M, Sinclair D. Sirtuin activators mimic caloric restriction and delay ageing in metazoans. Nature. 2004; 430:686–689.

53. Valenzano DR, Terzibasi E, Genade T, Cattaneo A, Domenici L, Cellerino A. Resveratrol prolongs lifespan and retards the onset of age-related markers in a short-lived vertebrate. Curr Biol. 2006; 16:296–300.

54. Rascón B, Hubbard BP, Sinclair DA, Amdam GV. The lifespan extension effects of resveratrol are conserved in the honey bee and may be driven by a mechanism related to caloric restriction. Aging (Albany NY). 2012; 4:499–508.

55. Pearson KJ, Baur JA, Lewis KN, Peshkin L, Price NL, Labinskyy N, Swindell WR, Kamara D, Minor RK, Perez E, Jamieson HA, Zhang Y, Dunn SR, et al. Resveratrol delays age-related deterioration and mimics transcriptional aspects of dietary restriction without extending life span. Cell Metab. 2008; 8:157–168.

56. Skulachev VP, Anisimov VN, Antonenko YN, Bakeeva LE, Chernyak BV, Erichev VP, Filenko OF, Kalinina NI, Kapelko VI, Kolosova NG, Kopnin BP, Korshunova GA, Lichinitser MR, et al. An attempt to prevent senescence: a mitochondrial approach. Biochim Biophys Acta. 2009; 1787:437–461.

57. Engel N, Mahlknecht U. Aging and anti-aging: unexpected side effects of everyday medication through sirtuin1 modulation. Int J Mol Med. 2008; 21:223–232.

58. Eisenberg T, Knauer H, Schauer A, Büttner S, Ruckenstuhl C, Carmona-Gutierrez D, Ring J, Schroeder S, Magnes C, Antonacci L, Fussi H, Deszcz L, Hartl R, et al. Induction of autophagy by spermidine promotes longevity. Nat Cell Biol. 2009; 11:1305–1314.

59. Saul N, Pietsch K, Menzel R, Stürzenbaum SR, Steinberg CE. The longevity effect of tannic acid in Caenorhabditis elegans: Disposable Soma meets hormesis. J Gerontol A Biol Sci Med Sci. 2010; 65:626–635.

60. Cañuelo A, Gilbert-López B, Pacheco-Liñán P, Martínez-Lara E, Siles E, Miranda-Vizuete A. Tyrosol, a main phenol present in extra virgin olive oil, increases lifespan and stress resistance in Caenorhabditis elegans. Mech Ageing Dev. 2012; 133:563–574.

61. Evason K, Collins JJ, Huang C, Hughes S, Kornfeld K. Valproic acid extends Caenorhabditis elegans lifespan. Aging Cell. 2008; 7:305–317.
